# Supplementary material for: An ancestral fold reveals the evolutionary link between RNA polymerase and ribosomal proteins
Source: Nat Commun. 2024 Jul 18;15:5938. doi: 10.1038/s41467-024-50013-9 (PMC11258233; doi:10.1038/s41467-024-50013-9)
Supplement: Supplementary file 1 — Supplementary Information [file 41467_2024_50013_MOESM1_ESM.pdf]

Supplementary information

An ancestral fold reveals the evolutionary link between RNA polymerase and ribosomal proteins

Sota Yagi[1,2*] and Shunsuke Tagami[1,3,4*]

Affiliations:

[1]RIKEN Center for Biosystems Dynamics Research, 1-7-22 Suehiro-cho, Tsurumi-ku, Yokohama, Kanagawa 230-0045, Japan

[2]Faculty of Human Sciences, Waseda University, 2-579-15, Mikajima, Tokorozawa, Saitama 359-1192, Japan.

[3]Graduate School of Medicine, Science and Technology, Shinshu University, 3-1-1 Asahi, Matsumoto City, Nagano, 390-8621, Japan

[4]International Institute for Sustainability with Knotted Chiral Meta Matter (WPI-SKCM²), Hiroshima University, 1-3-1 Kagamiyama, Higashi-Hiroshima, Hiroshima 739-8526, Japan

*Corresponding Authors: S.Y. (sota.yagi@aoni.waseda.jp) and S.T. (shunsuke.tagami@riken.jp)

This PDF file includes:

Supplementary Text

Supplementary Figs. 1 to 21

Supplementary Tables 1 to 5

Supplementary notes

1. Determination of the crystal structures of design-1 (third crystal type) and design-2

The structures of the third type of crystal of design-1 and design-2 could not be solved by molecular replacement with the DPBB structure. AlphaFold2 (AF2)[1] predicted that design-1 adopts the homo-dimeric DPBB fold like the 1st and 2nd crystal structures of design-1 (PDBIDs: 7DXY and 7DXZ). However, design-2 was predicted to form a homo-dimeric structure distinct from DPBB (Supplementary Fig. 3). The 1st loop formed a β-turn with a relatively low pLDDT. Furthermore, the 2nd β-strands were inverted so that all six β-strands in the dimer were anti-parallel. These structural features are consistent with the RIFT fold[2]. Using this RIFT structure as the model for molecular replacement, we solved the structures of the third type of crystal of design-1 and design-2. All of the barrel structure strands fitted well to the electron density maps. However, we needed to manually remodel the 1st loop because it showed a rolled-up loop configuration that was completely different from the β-turn in the AF2 model. We named this newly found fold the Double Zeta Beta Barrel (DZBB).

Perhaps the crucial factor for the molecular replacement was the similarity of the β-barrel part, which is all anti-parallel. In contrast, the wrongly predicted 1st loop was tolerated in the molecular replacement process. Although AF2 is renowned for its high accuracy, we needed to carefully remodel the predicted structures as such errors are still not rare. In particular, its prediction ability for novel structures, like the DZBB fold, that have not been registered in databases may be limited.

We also found that the chemically synthesized design-1, which does not include the five "GPMPG" residues at the N-terminus, also formed the homo-dimeric DZBB structure under the same crystallization conditions (Supplementary Fig. 5A and Supplementary Table 3). Therefore, regardless of the short N-terminal tag, the 43 aa sequence in design-1 can adopt two homo-dimeric β-barrel structures, DPBB and DZBB.

2. Several anions facilitate the folding of design-1

We verified that high concentrations of malonate induced the conformational

change of design-1 (Fig. 2C). We also tested if the other carboxylic acids could mediate the folding of design-1. In the previous study, design-1 was also crystalized with the DPBB structure in the presence of a high concentration of malate[3]. As expected, 2M malate also promoted the conformational change (Supplementary Fig. 10A). Likewise, 2M citrate also changed the fluorescence spectra in a similar manner to malonate and malate (Supplementary Fig. 10B), although significant increments of ANS fluorescence were not observed using acetate, formate, and glycine (Supplementary Fig. 10C–E). Thus, among the carboxylic acids, di- or tri-carboxylic acids (but not mono-carboxylic acids) were most likely to promote the folding of design-1 into the DPBB fold.

We also tested a high phosphate ion concentration, as its tetrahedral structure and anionic properties are similar to those of sulfate, which likely induced folding into the DZBB fold. ANS and CD experiments verified that 2M phosphate, like sulfate, also enhances the folding of design-1, most likely into DZBB (Supplementary Fig. 9C and 10F).

These results indicated that the simplified ancestral peptide, design-1 adopts two different homo-dimeric folds, DPBB and DZBB, under different conditions (Figs. 1 and 2). This dual-folding property is thought to represent a relic during an early fold transition event; i.e., the innovation process that still retains the ancestral conformational state while transitioning to a new conformation[4–6]. Notably, the fold conversion of design-1 depends on the types of small ligands (Figs. 2 and Supplementary Fig. 10) that are universally present in living organisms. Malate and malonate, which induce design-1 to form DPBB, are a substrate and an inhibitor, respectively, in the most ancient metabolic pathway, the tricarboxylic acid cycle. In contrast, high concentrations of sulfate, and probably phosphate as well, lead design-1 to fold into DZBB. These small negative ions are also essential compounds and resources for the biosynthetic pathways of sulfur-containing amino acids, nucleotides, and coenzymes[7,8] (for example, CoA). Thus, these compounds were likely central materials in the metabolic systems of primitive life and might also have worked as fold-evolution triggers.

3. Stability enhancement by sulfate ion

Sulfate ions were also observed in the positively charged pockets of the crystal structures of design-2 (DZBB-fold), design-3, and design-4 (RIFT-fold) (Fig. 1 and

Supplementary Fig. 8). To examine if sulfate ions enhanced the folding and stability of these proteins, we performed CD experiments. The thermal stability of design-2 was moderate (Tm = 58˚C). However, in the presence of 500 mM ammonium sulfate, it mostly remained in the native structure even at 70˚C, and could be refolded after heat denaturation (Supplementary Fig. 4E). In addition to their thermal stabilities, the secondary structure contents of design-2, design-3, design-4, and design-5 were increased by adding 500 mM sulfate ion (Supplementary Fig. 7). Especially, although design-3 unfolded in the absent of sulfate, the addition of sulfate significantly mediated its folding (Supplementary Fig. 7C). Therefore, the sulfate ion might serve as a chemical chaperone to enhance the folding of ancient proteins with less stability. Likewise, other groups have recently reported folding stabilization and enhancement of reconstructed proteins by magnesium ions, sodium chlorides, and polyamines[9–11]. Therefore, instead of focusing only on the sequence of the protein molecule, exogenous variables like ion types and concentrations should be considered simultaneously when investigating ancient proteins.

4. Experimental conversion from the DZBB fold to the OB fold

1st generation of DZBB-OB chimeras

The monomeric part of design-1 (DZBB) superimposed well onto the OB-domain in rL2 derived from *Thermococcus kodakarensis*, with a 2.4 Å RMSD value (Fig. 3A). Based on the superimposed structures, the sequences of design-1 and the OB-domains of rL2 from *T. kodakarensis* and *Methanopyrus kandleri* were aligned (Supplementary Fig.11A). The conserved sequence motif [G/A]xV in β1 of the OB-fold is present in the design-1. The GD box motif commonly conserved in the α1–β3 loop of the DPBB-, RIFT-, and DZBB-folds is also present in the OB-fold.

To convert DZBB to the OB-fold, we engineered design-1 by replacing the sequence between the C-terminus of β1 and the C-terminus of β2 or α1 with the corresponding sequences in the OB domain from *T. kodakarensis* and *M. kandlerii* rL2, yielding six chimeric proteins: design-6 to -11 (tkoL2_v1, mkaL2_v1, tkoL2_v2, mkaL2_v2, tkoL2_v3, and mkaL2_v3)(Supplementary Fig. 11B). As rL2 does not have α1, the designs excluding α1 of design-1 (design-6 and design-9) were expected to have all-β structures. In contrast, the other four chimeras retained the α1 sequence from design-1. The difference between design-7 (or design-10) and design-8 (or design-11) is the joint

region between design-1 and OB from rL2 (Fig. S10B). In design-7 and design-10, the OB segment is connected to the design-1 segment, so it does not disturb the boundary structures of the superimposed models. In the cases of design-8 and design-11, the connection site was designed simply based on the sequence alignment. All designs were expressed well, but only design-6 and design-9 folded (Supplementary Fig. 12).

2nd generation of DZBB-OB chimeras

We also constructed design-12 and design-15 by combining the OB-domain of rL2 and design-2, as in design-6 and design-9 (Supplementary Fig. 13A). Both mutants exhibited similar folding properties to design-6 and design-9 (Supplementary Fig. 14A and D). Crystallographic analysis confirmed that design-9 adopted the four stranded OB-fold (Supplementary Fig. 5D). These chimeras were subsequently modified to resemble design-2 more closely, by replacing the residues within the β3 regions with their corresponding residues in design-2 (Supplementary Fig. 13A). The resultant mutants design-13 and design-16 were predicted by AF2 to retain the OB-fold conformation. Crystallographic analysis confirmed that design-13 adopted the OB-fold (Fig 3E).

In the sequence alignment of these designs with design-2, they share the patterns of charged residues, x-R-x-E-D-x-x, in the Z-loop-β2 region of design-2 (Supplementary Fig. 13A). Thus, we subsequently designed design-14 and design-17 by introducing the design-2 residues within this region (Supplementary Fig. 13A). However, design-14 was unfolded, and design-17 could not be purified due to its low solubility (Supplementary Fig. 14). The introduced mutations, especially F28A in the hydrophobic core might have destabilized the OB structure. Therefore, design-13 and design-16 were the DZBB-OB intermediates with the most stable OB structures and the highest identity (59%) with design-2 (DZBB-fold) (Supplementary Fig. 13A).

3rd generation of DZBB-OB chimeras

The long β1–β2 loop in the DZBB-OB chimeras designed above is likely flexible, as its configuration is variable in their crystal structures (Supplementary Fig. 16A). Additionally, in native OB-fold proteins, the corresponding loop regions are highly diverse, and some possess short β-turns instead of the long loop. Thus, to simplify the DZBB-OB chimeras, the 8 a.a. residues within the β1–β2 loops of design-6, design-13,

and design-16 were replaced with 2 a.a. residues optimized to form an ideal β-turn, resulting in design-20, design-21, and design-22 (Supplementary Fig. 16B). These mutants were soluble, folded, and had comparable thermal stabilities to their parents (Supplementary Fig. 17). Additionally, we determined the crystal structure of design-20 and confirmed that the engineered β1–β2 loop adopted a short β-turn (Supplementary Fig. 16C). The a.a. sequences of design-22 (OB-fold) and design-19 (DZBB) are 77 % identical. The remaining different residues (7 a.a. in/del and 3 a.a. point mutations) should be the determinants for the fold transitions between OB- and DZBB-folds.

In the SEC experiment of design-22, we detected two peaks corresponding to the molecular masses of the monomer and homo-dimer (Supplementary Fig. 17C). Although other DZBB-OB chimera proteins were monomers in solution, design-6, design-9, design-15, and design-20 formed pseudo-homo-dimeric structures by crystal packing (Supplementary Fig. 18A–D). In their crystal structures, the two monomers bound to each other by the exposed hydrophobic residues at the partial slit of the barrel. An intermolecular β-sheet was also formed between the β4 strands of the two monomers. A similar anti-parallel β-sheet at the subunit interface was observed at the homo-dimeric interface of the DZBB structure (Supplementary Fig. 18E). Therefore, design-22 probably forms a symmetric homo-dimer in solution in a similar manner. Such homodimerization of a four-stranded OB-fold might have been an intermediate step during the ancient fold transitions between DZBB- and OB-folds.

5. The evolutionary pathways between ancient barrels

DPBB vs DZBB vs RIFT

As the DPBB and RIFT folds have similar six-stranded β-barrel structures with two-fold internal pseudo-symmetry and share detectable sequence similarity (Figs 1 and Supplementary Fig. 2), they were assumed to have evolved from a common ancestral protein. Two hypotheses can explain the evolutionary pathway between DPBB and RIFT. The first hypothesis is that the ancient homo-dimeric RIFT protein evolved into a monomeric protein by gene duplication and fusion[2,12]. This monomeric RIFT then gave rise to monomeric DPBB by swapping of second and fourth β-strands. The second hypothesis is that the original ancient peptide diverged into homo-dimeric DPBB and RIFT, and the gene duplication and fusion of the homo-dimeric DPBB and RIFT

independently evolved into monomer proteins[3,13]. We previously showed that short 43 a.a peptides could adopt the homo-dimeric DPBB structure with high stability[3]. Furthermore, the current study demonstrated that the homo-dimeric DPBB could convert into the DZBB fold and then into the homo-dimeric RIFT fold (Figs. 1 and 5). These experimental results strongly support the second evolutionary hypothesis, with the hidden evolutionary route through DZBB.

DZBB vs OB

Recently, Loren William's group proposed the "creative destruction" hypothesis to explain how distinct protein folds diverged[14,15]. In this hypothesis, the gene encoding the ancestral protein was duplicated and fused tandemly. Then, some extraneous secondary elements were lost while maintaining some elements in the daughter fold. They found the sequence similarity between OB and RIFT and then applied this hypothesis to interpret its fold transition (OB→tandem OB→RIFT). However, the adaptation process from the tandem OB fold to the RIFT fold is complicated and requires multiple sequence deletion events, with intermediates that have not been shown to be foldable or functional.

In contrast, the novel homo-dimeric DZBB fold obviously shared structural similarities with the OB-fold proteins. In addition, the chimera-genesis and sequential mutation experiments showed that DZBB could be converted into OB through a few point mutations and a single short insertion (Fig. 4 and Supplementary Figs. 11–17). Considering the evolutionary relationship between the RIFT and DZBB folds (Fig. 1), the homo-dimeric RIFT fold was likely altered into the OB fold via the homo-dimeric DZBB fold, without gene duplication (Fig. 5). As a few mutations and short In/Dels suffice for the RIFT-DZBB-OB fold conversions, this evolutionary pathway is a more feasible scenario.

OB vs SH3

The reconstructed OB-fold protein possesses a presumed ancestral structural property, a four-stranded barrel structure[16]. Furthermore, by rearrangement of the order of the β-strands in the OB-fold protein archetype to mimic circular permutation, we succeeded in reconstructing the four-stranded SH3 fold (Fig 3G). While the structural similarities between the OB and SH3 fold and their divergent evolution have been debated over the

last three decades, our results are the first experimental verification of their transition, strongly supporting the recent hypothesis that the ancestral four-stranded OB fold evolved to the four-stranded SH3 fold by the simple circular permutation of a single β-strand (or vice versa)(Fig. 5)[16].

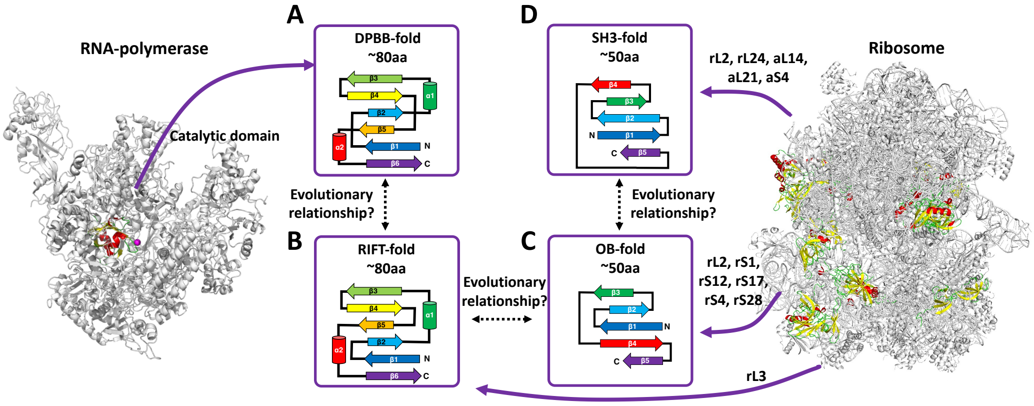

Supplementary figure 1 The small β-barrel folds conserved in the RNA polymerase and the ribosome. (A) The catalytic domain of the RNA polymerase is composed of two DPBB domains. Several ribosomal proteins have the conserved (B) RIFT-, (C) OB-, and (D) SH3-folds. The evolutionary relationships between DPBB–RIFT, RIFT–OB, and OB–SH3 pairs have been suggested in earlier studies[2,12,15,17].

A

B

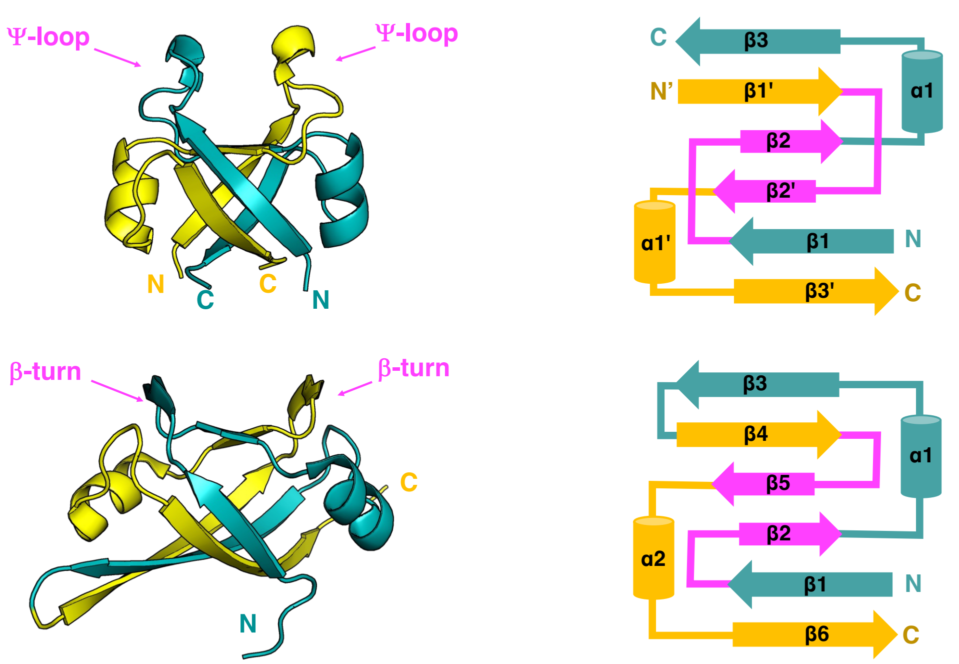

C

```
Design-1          MPGKKVVARVAEARAEDVGKRVVKVDKAERAKVGVKVGDVVEVKKV
Phs018_Nhalf     MDVLAKFHTTVHR------IG--RIIIPAGTRKFYGIEQGDFVEIKIVKYEG
Phs018_Chalf    EEPKEGTFTARVGE------QG--SVIIPKALRDVIGIKPGEVIEVLLLGHYKPRN
                       ▲                ▲▲▲          ▲
```

D

	Design-1	Phs018_Nhalf	Phs018_Chalf
Design-1		53%	47%
Phs018_Nhalf	35%		52%
Phs018_Chalf	26%	25%	

Supplementary figure 2. Comparison of the DPBB- and RIFT-folds. (A) The structure of the homo-dimeric DPBB, design-1. Each chain is colored cyan and yellow. (B) Structure of the presumed ancestral RIFT protein, Phs018 (PDBID: 2GLW). Its pseudo-repeat units are colored cyan and yellow. The significant differences in structures between DPBB and RIFT are highlighted in pink. (C) Sequence alignment of the design-1 and the N- and C-halves of Phs018. Completely and partially conserved residues are highlighted in red and pink, respectively. The residues specifically conserved in Phs018 are marked with black triangles. (D) Identity and homology between sequence pairs were calculated based on the alignment of Fig. S2C and are shown in the left-bottom and right-top corners, respectively.

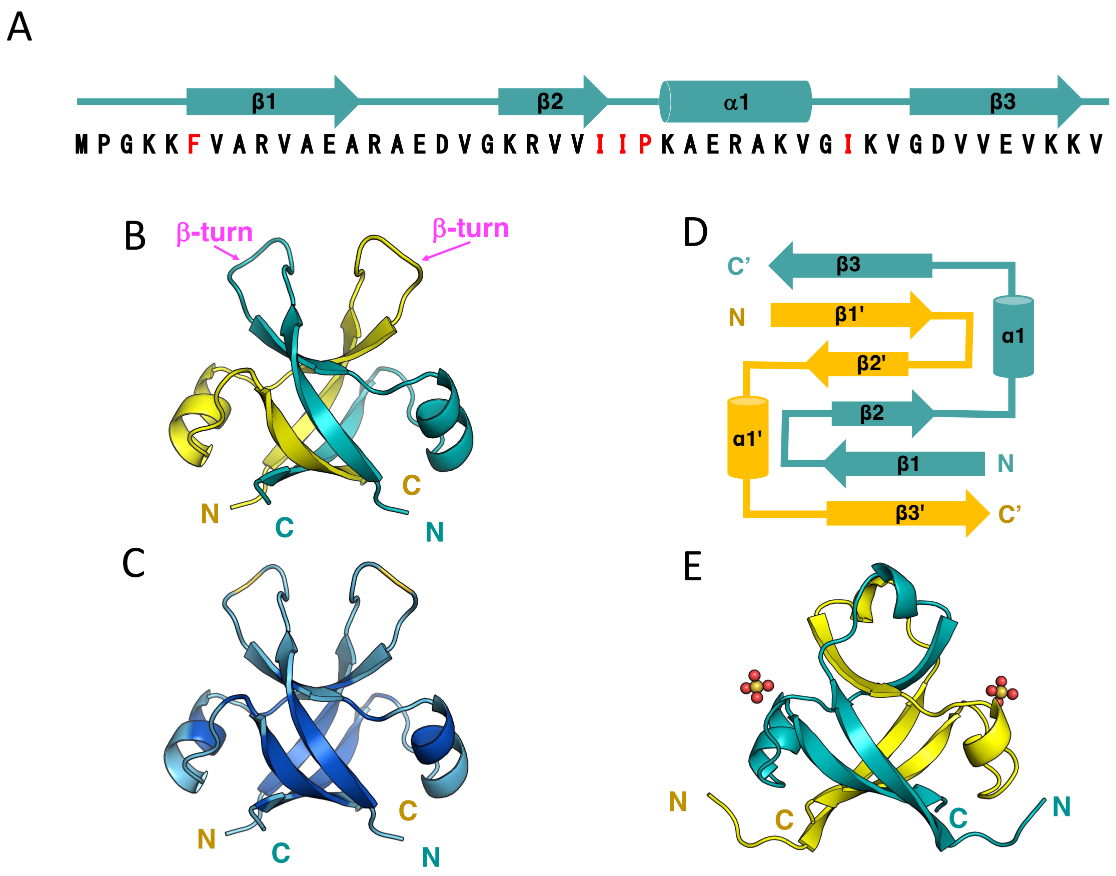

Supplementary figure 3. Design of the design-2. (A) The amino acid sequence of design-2. The five amino acids introduced into design-1 are colored red. (B and C) The structure of design-2 predicted by AlphaFold2. (C) Based on the pLDDT values in the prediction of design-2, each amino acid is colored red (pLDDT=0) to blue (pLDDT=100). The 1st loop forming the β-turn "16E–18V" exhibited a relatively lower pLDDT value (72–65) than the other region. (D) Topology of the predicted design-2. (E) The crystal structure of design-2 replicated from Fig. 1D.

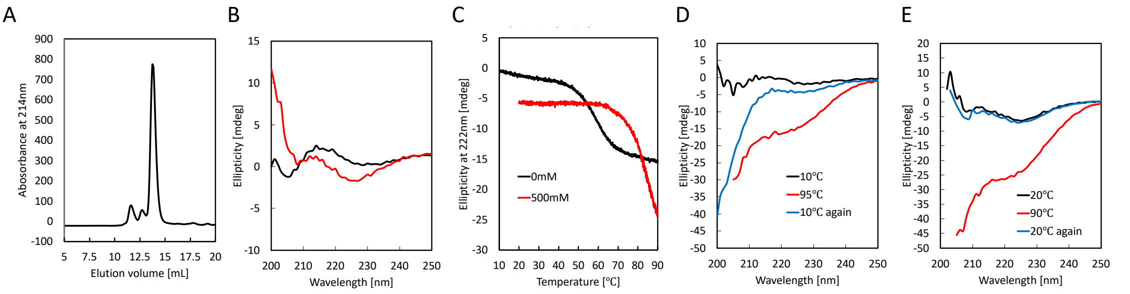

Supplementary figure 4. Experimental characterization of design-2. (A) Size exclusion chromatogram. (B) CD spectra in the absence and presence of 500 mM ammonium sulfate, shown as black and red lines, respectively. (C) Melting curves of design-2 in the absence and presence of 500 mM ammonium sulfate, shown as black and red lines, respectively. (D and E) CD spectra of design-2 in the absence and presence of 500 mM ammonium sulfate at different temperatures (black: 10 or 20ºC; red: 90ºC; blue (refolding): 90ºC -> 10 or 20ºC). In the CD experiment, the protein concentration was 13 μM. Source data are provided as a Source Data file.

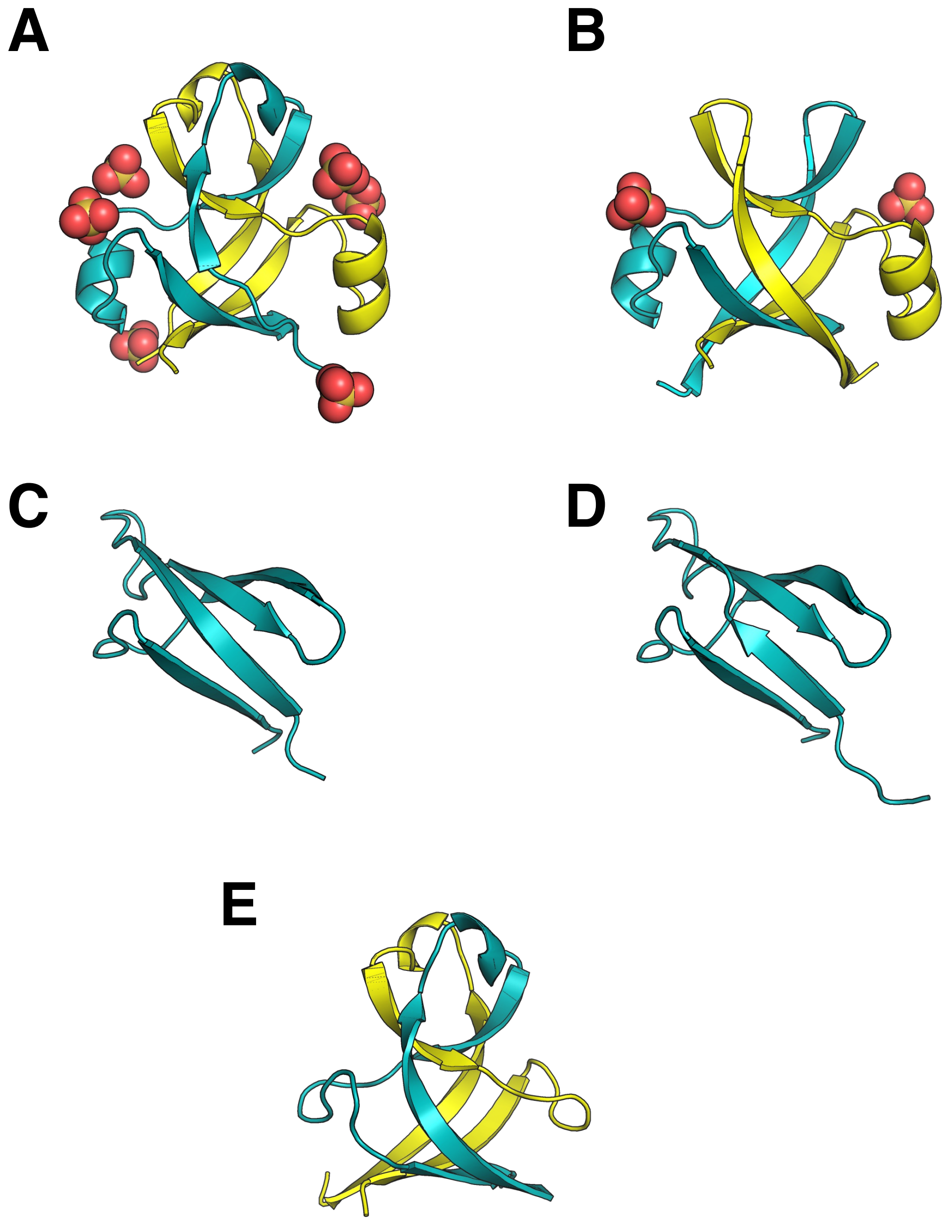

Supplementary figure 5. Crystal structures of the (A) the chemically synthesized design-1 with sulfate ions, (B) design-4 with sulfate ions, (C) design-9, (D) design-15, and (E) design-19.

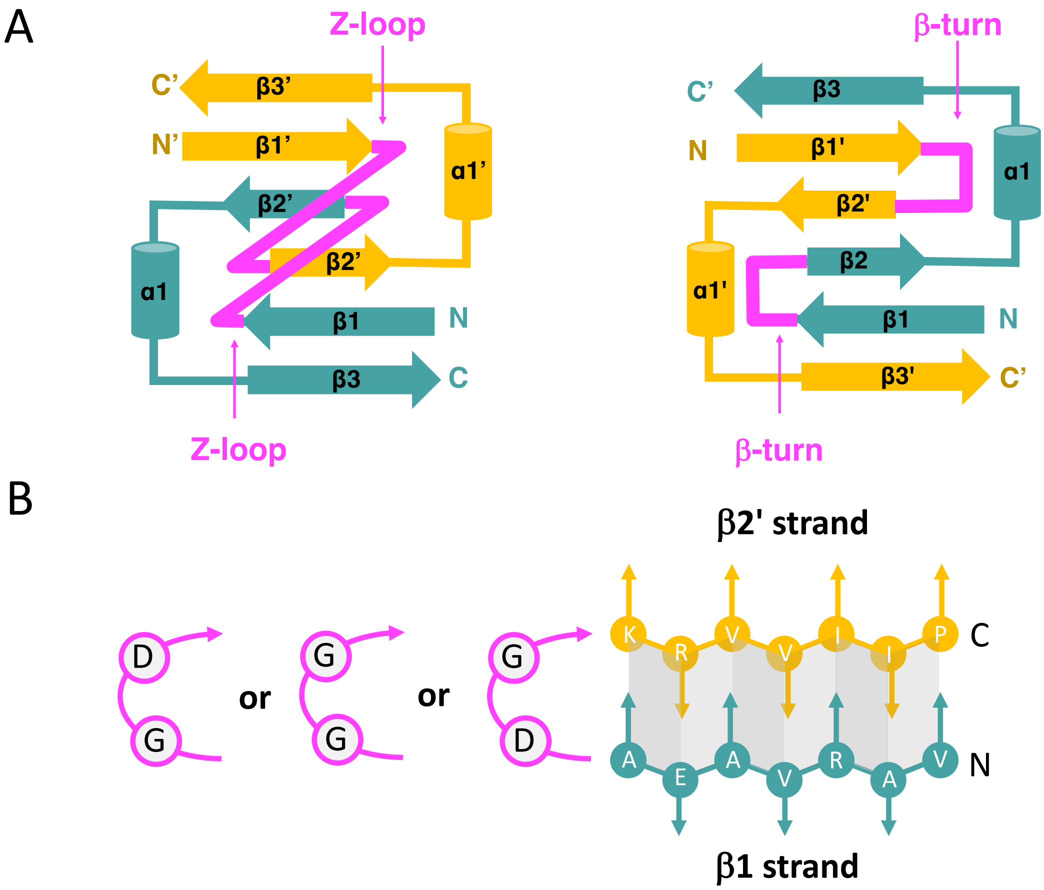

Supplementary figure 6. Design of the homo-dimeric RIFT fold. (A) Comparison of the topologies of the DZBB and RIFT folds. The 1st loops, colored pink, are the main different structures between the two folds. (B) The 1st loop, forming a Z-loop in the DZBB protein, was engineered so that it forms a β-turn with the 2 a.a. sequence “DG”, “GG”, or “GD.”

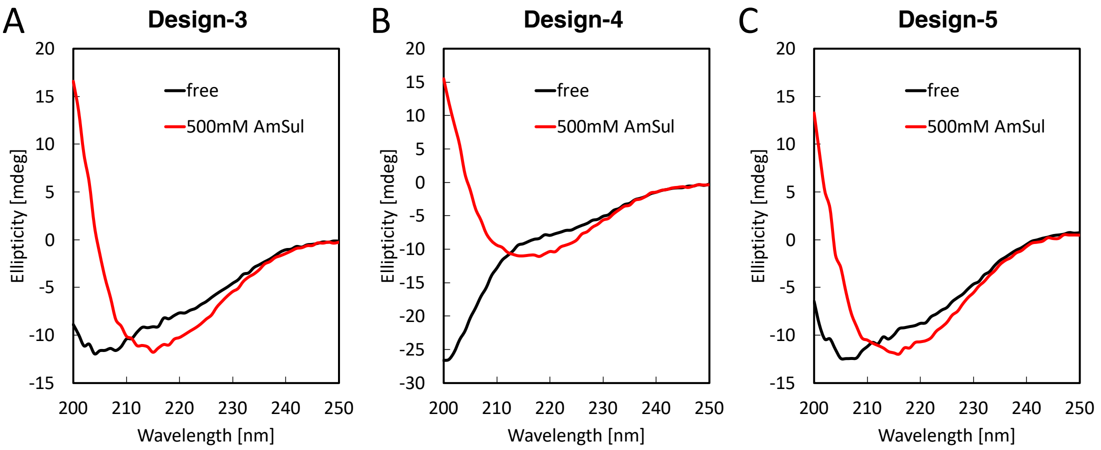

Supplementary figure 7. CD spectra of the designed homo-dimeric RIFT proteins. The spectra for design-3 (A), design-4 (B), and design-5 (C) were measured in the absence (black line) or presence (red line) of 500 mM ammonium sulfate. Source data are provided as a Source Data file.

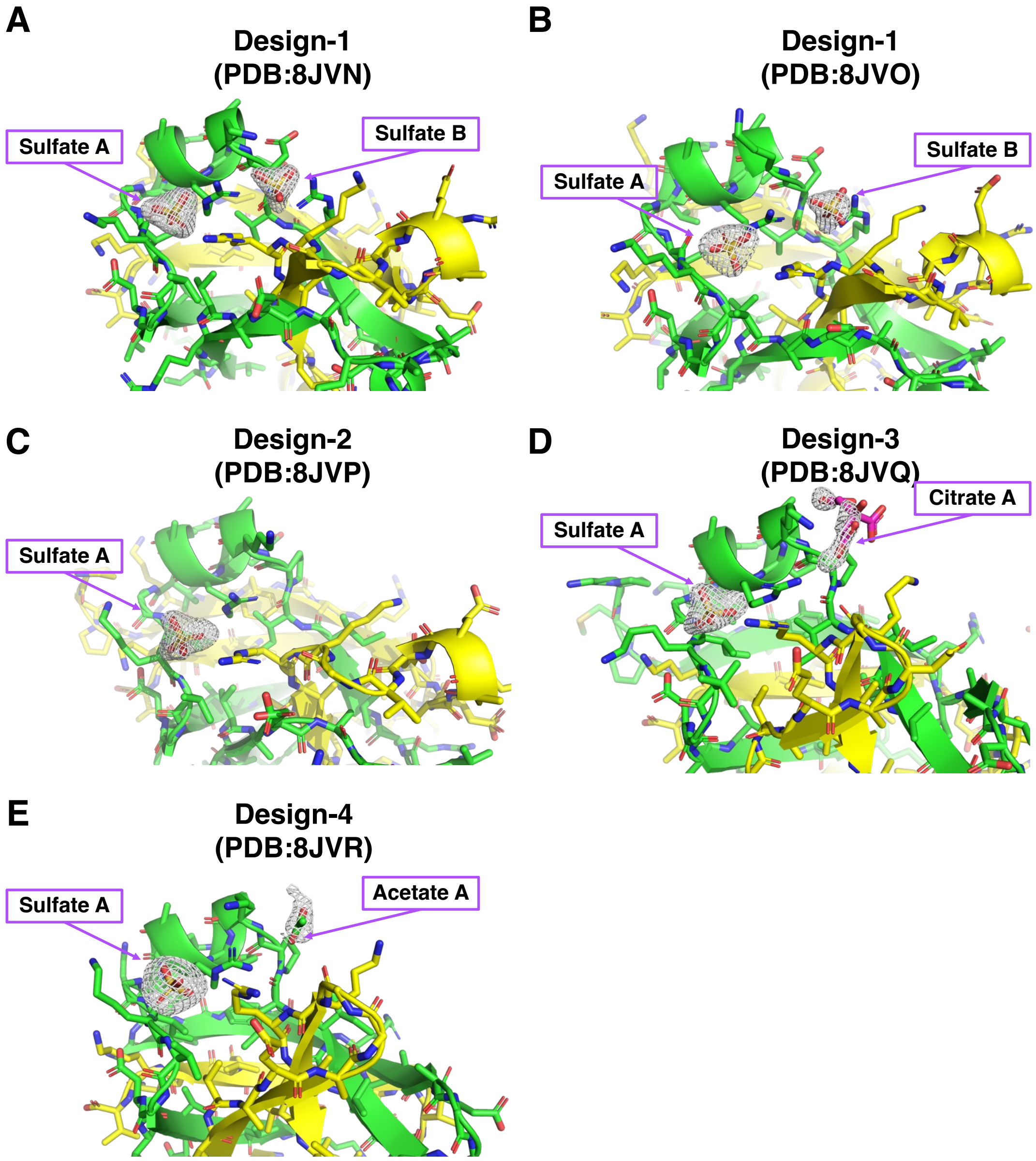

Supplementary figure 8. The omit map density of ligands from the design-1 (A and B), design-2 (C), design-3(D), and design-4 (E) are shown at contour levels of 3σ.

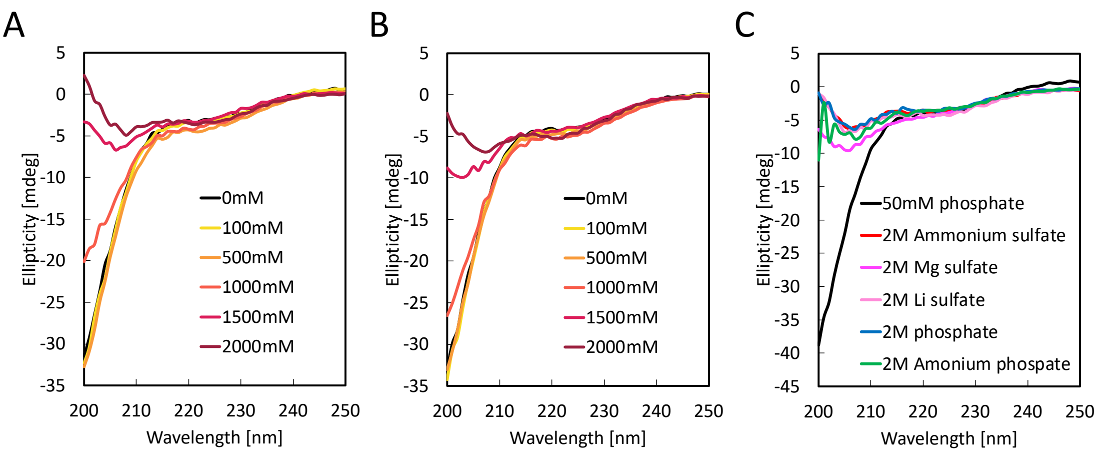

Supplementary figure 9. CD spectra of design-1 under different buffer conditions. The spectra of bacterially produced design-1 (A) and chemically synthesized design-1 (B) in various ammonium sulfate concentrations (0–2,000 mM). (C) The CD spectra in different high concentrations of sulfate or phosphate salts. Source data are provided as a Source Data file.

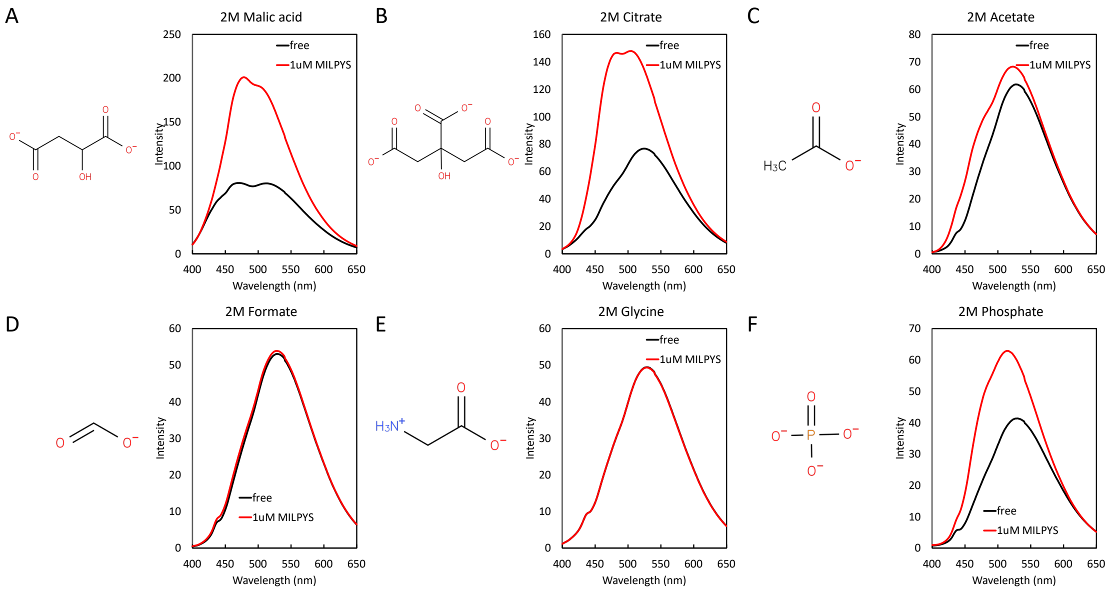

Supplementary figure 10. Fluorescence spectra of ANS with (A)2 M malic acid, (B) 2 M citrate, (C) 2 M acetate, (D) 2 M formate, (E) 2 M glycine, and (F) 2 M potassium/sodium phosphate. The emission spectra were recorded with excitation at 380 nm in the absence (black line) or presence (red line) of 1 µM design-1. Source data are provided as a Source Data file.

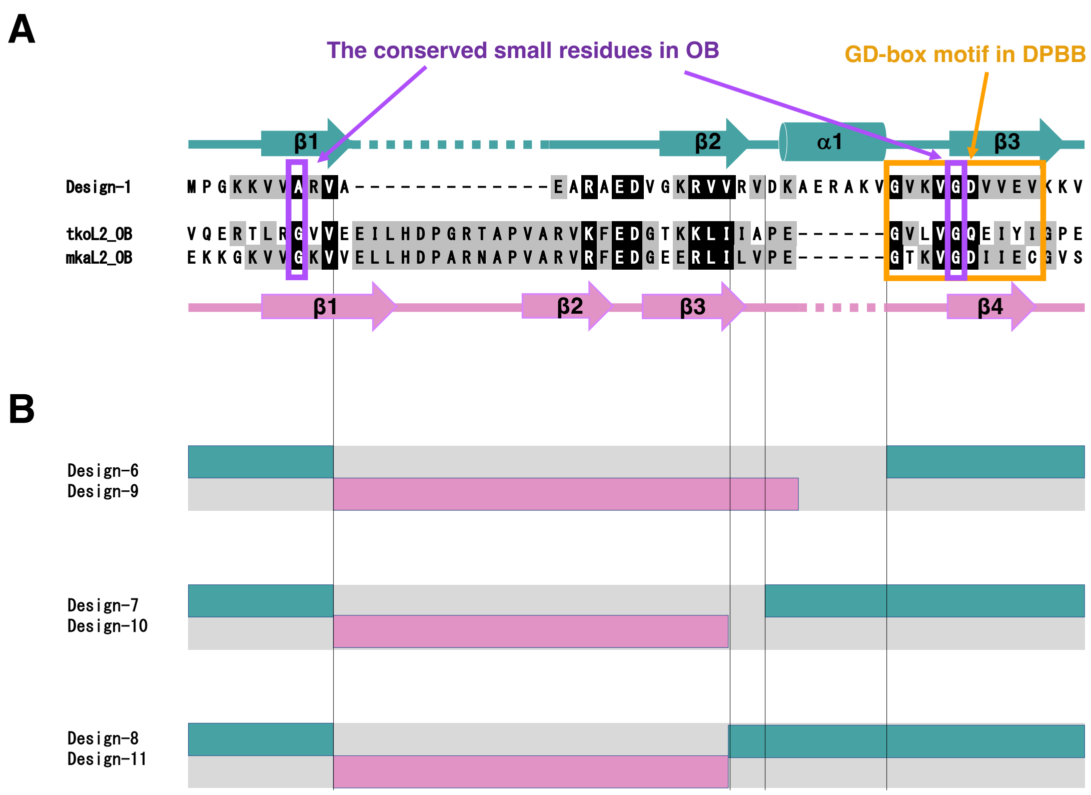

Supplementary figure 11. (A) Sequence alignment of design-1 and the OB domains in ribosomal protein L2 from *T. kodakarensis* and *M. kandleri*. The reported sequence motifs that are specifically conserved in OB- or DPBB-folds, are enclosed by purple or orange boxes, respectively[18,19]. The completely or partially conserved residues are colored black and gray, respectively. (B) The design of the 1st generation DZBB-OB chimera. We introduced the central parts of the rL2 OB-domain (pink bars) to the corresponding positions in design-1 (cyan bars). Different chimeras were constructed by changing the positions and extents of the joined segments.

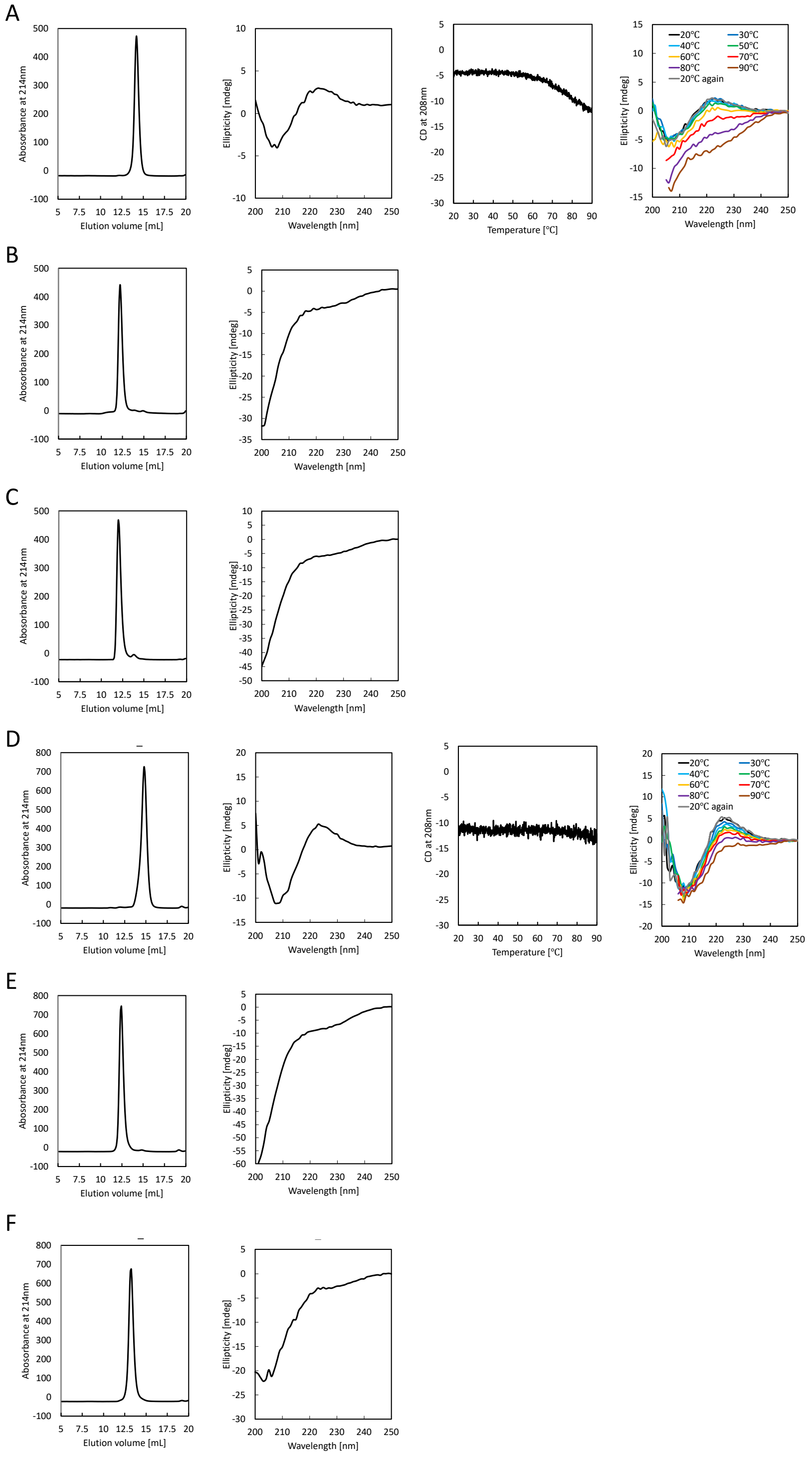

Supplementary figure 12. Experimental characterization of the 1st generation of OB-DZBB chimeras. Size exclusion chromatography, CD spectra, denaturation curves, and comparisons of CD spectra at different temperatures (20, 30, 40, 50, 60, 70, 80, 90, 20°C again (refold)) for (A) design-6, (B) design-7, (C) design-8, (D) design-9, (E) design-10, and (F) design-11. Source data are provided as a Source Data file.

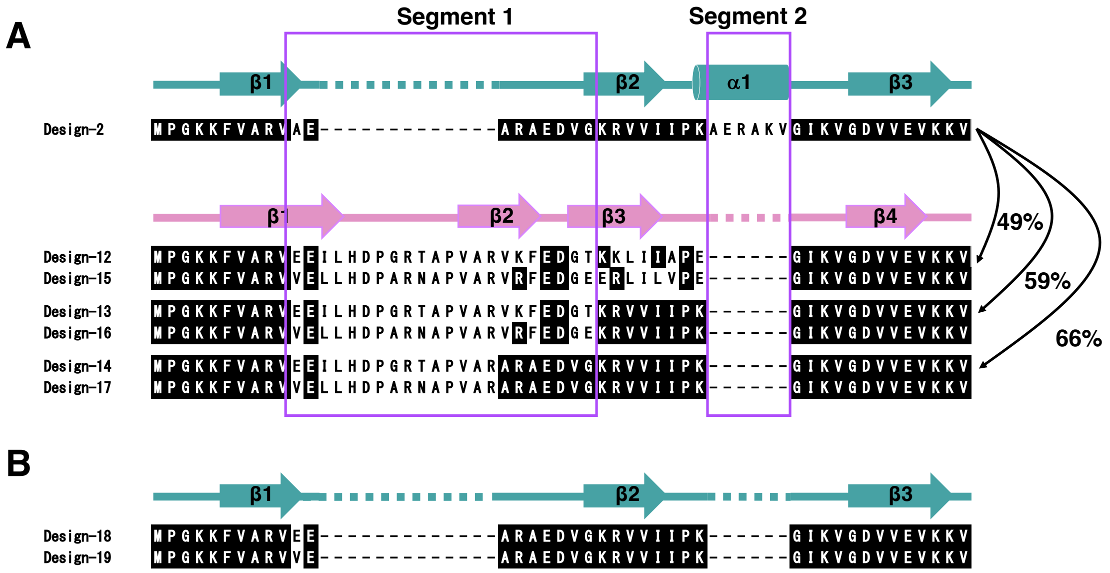

Supplementary figure 13. Design of the 2nd generation of DZBB–OB chimeras. (A) Sequence alignment of the chimeric proteins and design-2. The identical residues with design-2 are black, and the sequence identities between design-2 and each chimera are shown on the right. Pink boxes enclose the two significantly different segments between design-2 and design-13/ design-16. (B) The sequences of design-18 and design-19.

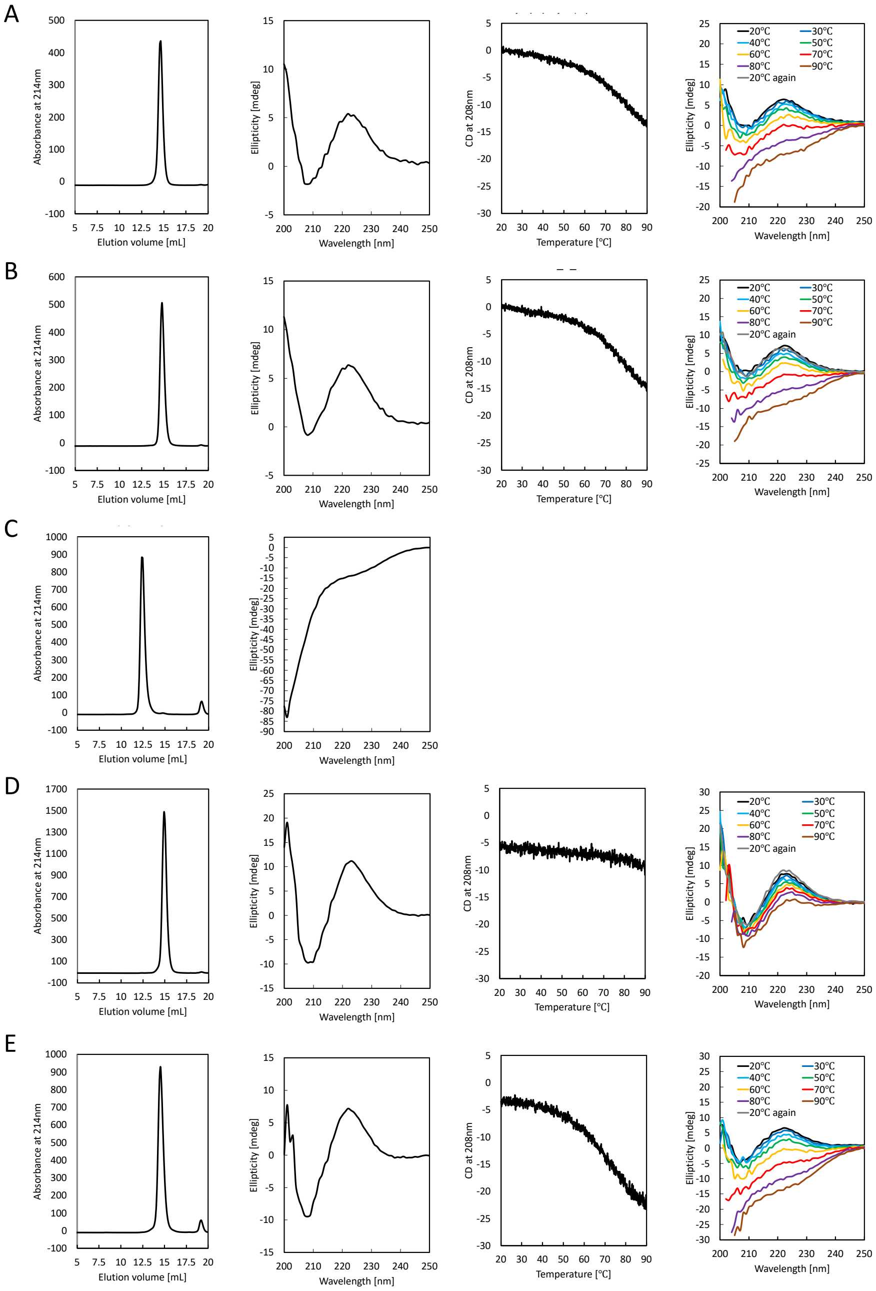

Supplementary figure 14. Experimental characterization of the 2nd generation of DZBB-OB chimeras. Size exclusion chromatography, CD spectra, denaturation curves, and comparisons of CD spectra at different temperatures (20, 30, 40, 50, 60, 70, 80, 90, 20°C again (refold)) for (A) design-12, (B) design-13, (C) design-14, (D) design-15, and (E) design-16. The concentrations of design-15 and design-16 were diluted to 13 µM in the CD experiment to reduce the high tension voltage at the shorter wavelengths. Source data are provided as a Source Data file.

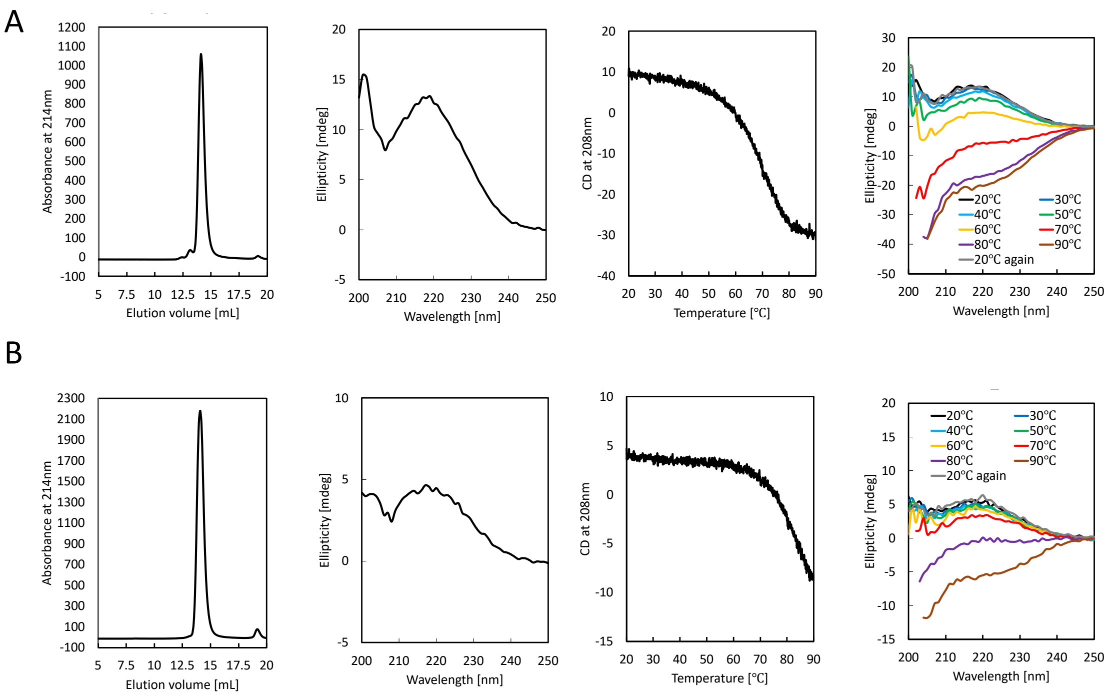

Supplementary figure 15. Experimental characterization of the reverse engineered DZBB-OB chimeras. Size exclusion chromatography, CD spectra, denaturation curves, and comparisons of CD spectra at different temperatures (20, 30, 40, 50, 60, 70, 80, 90, 20°C again (refold)) for (A) design-18 and (B) design-19. The concentrations of design-18 and design-19 were diluted to 13 and 2.5 μM, respectively, in the CD experiment to reduce the high tension voltage at the shorter wavelengths. Source data are provided as a Source Data file.

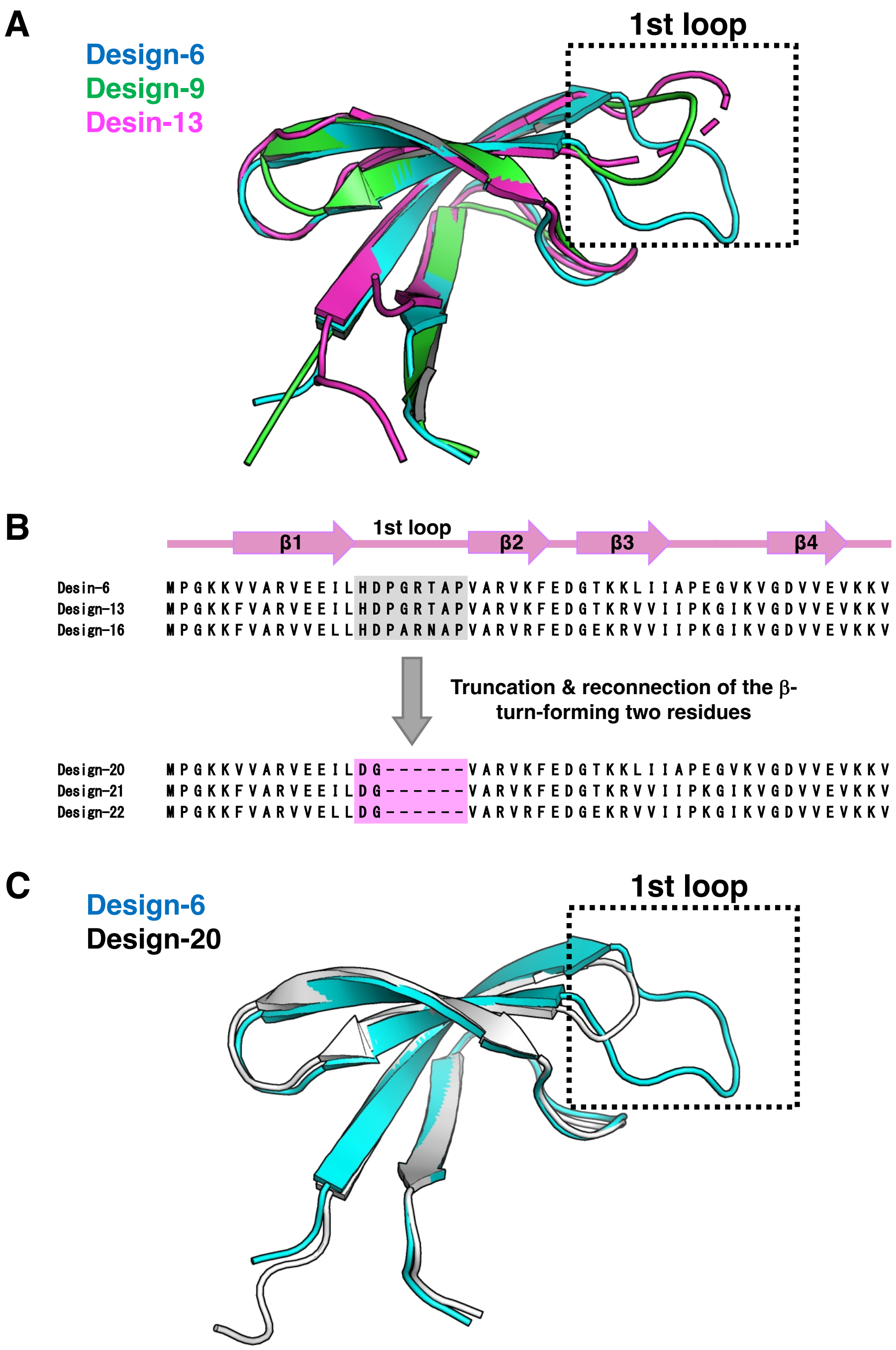

Supplementary figure 16. Truncation of the flexible loop in the DZBB-OB chimeras. (A) Superimposition of the crystal structures of design-6, design-9, and design-13. The configurations of the long 1st loop are significantly different. (B) The sequences in the 1st loop in the chimera were truncated and reconnected with two residues, “DG,” forming a short β-turn. (C) The crystal structure of design-20 is superimposed with its parent design-6. The engineered region in the design-20 formed the expected short β-turn.

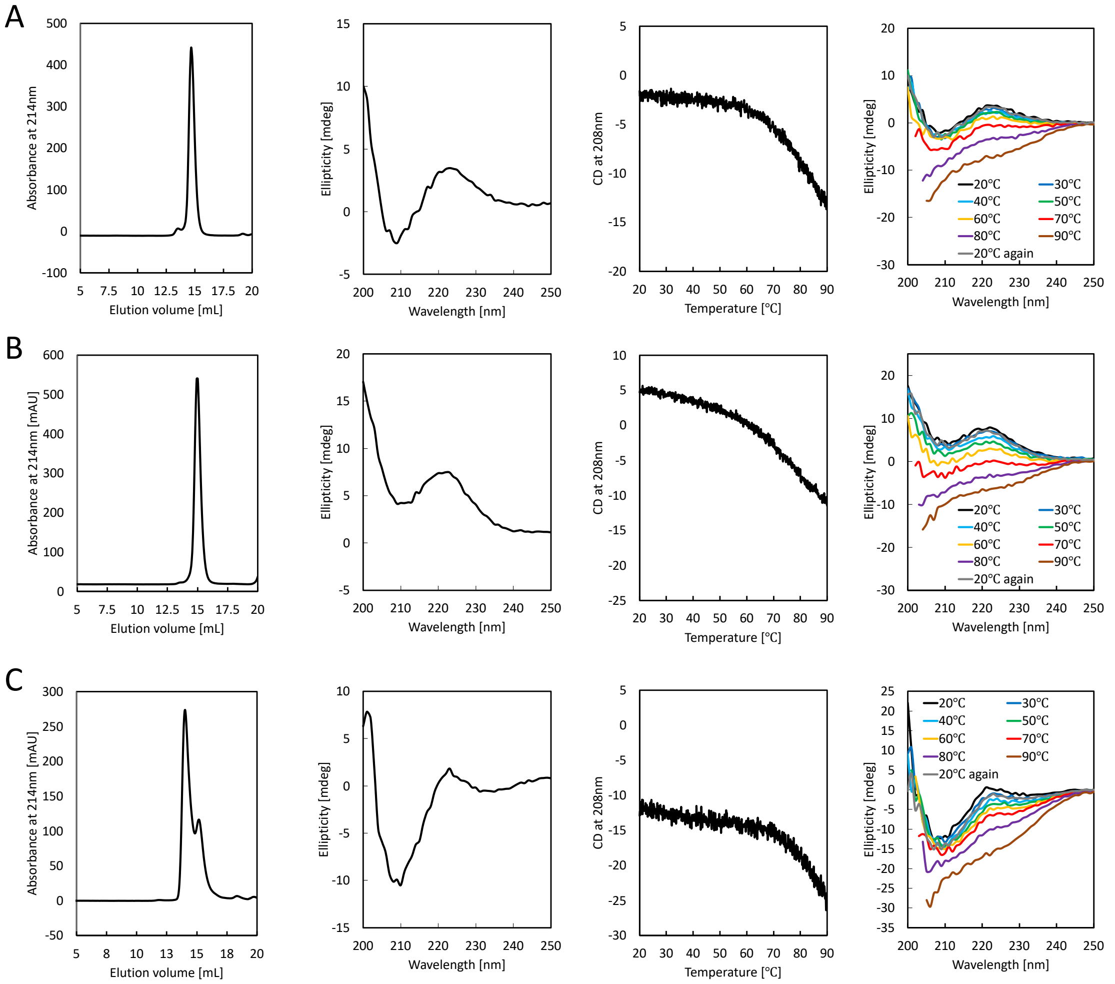

Supplementary figure 17. Experimental characterization of the 3rd generation of DZBB-OB chimeras. Size exclusion chromatography, CD spectra, denaturation curves, and comparisons of CD spectra at different temperatures (20, 30, 40, 50, 60, 70, 80, 90, 20°C again (refold)) for (A) design-20, (B) design-21, and (C) design-22. The concentration of mkaL2_v1.2_turn was diluted to10 µM in the CD experiment to reduce the high tension voltage at the shorter wavelengths. Source data are provided as a Source Data file.

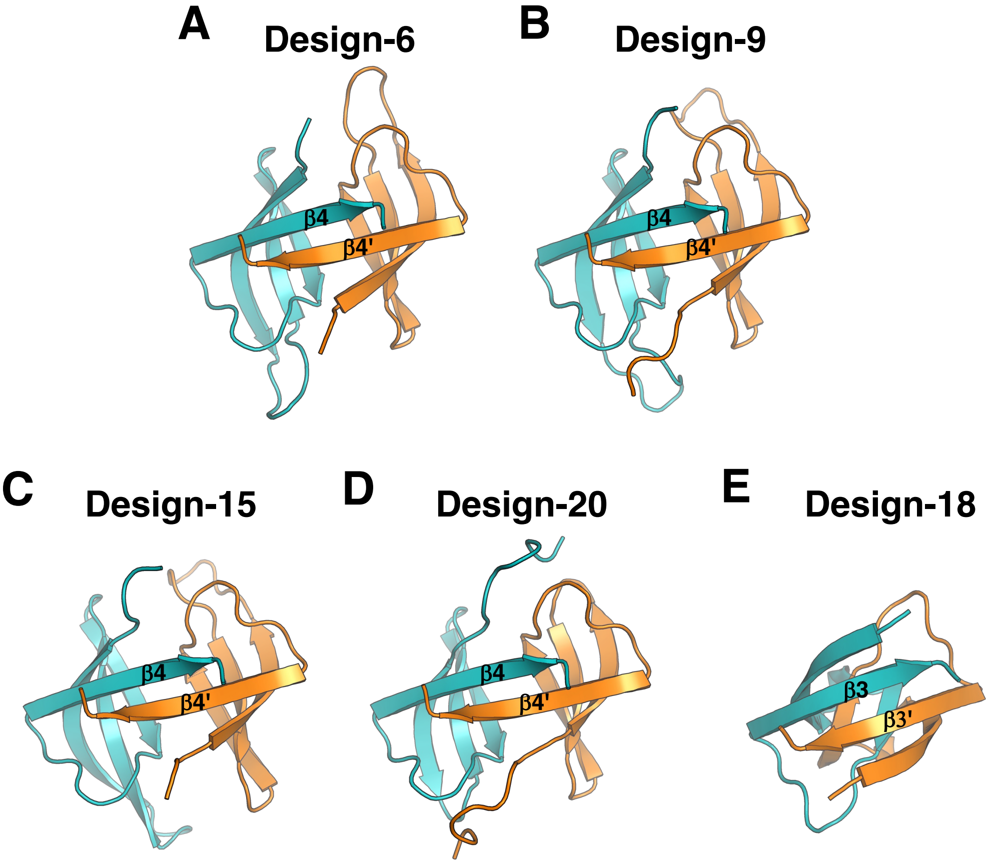

Supplementary figure 18. Pseudo homo-dimeric OB folds in the crystal structures. (A) design-6, (B) design-9, (C) design-15, and (D) design-20 formed pseudo homo-dimeric structures through the formation of an anti-parallel β-sheet with the 4th β-strands, although they existed as monomers in solution. (E) design-18 formed a stable homo-dimer by forming an anti-parallel β-sheet with the 3rd β-strands.

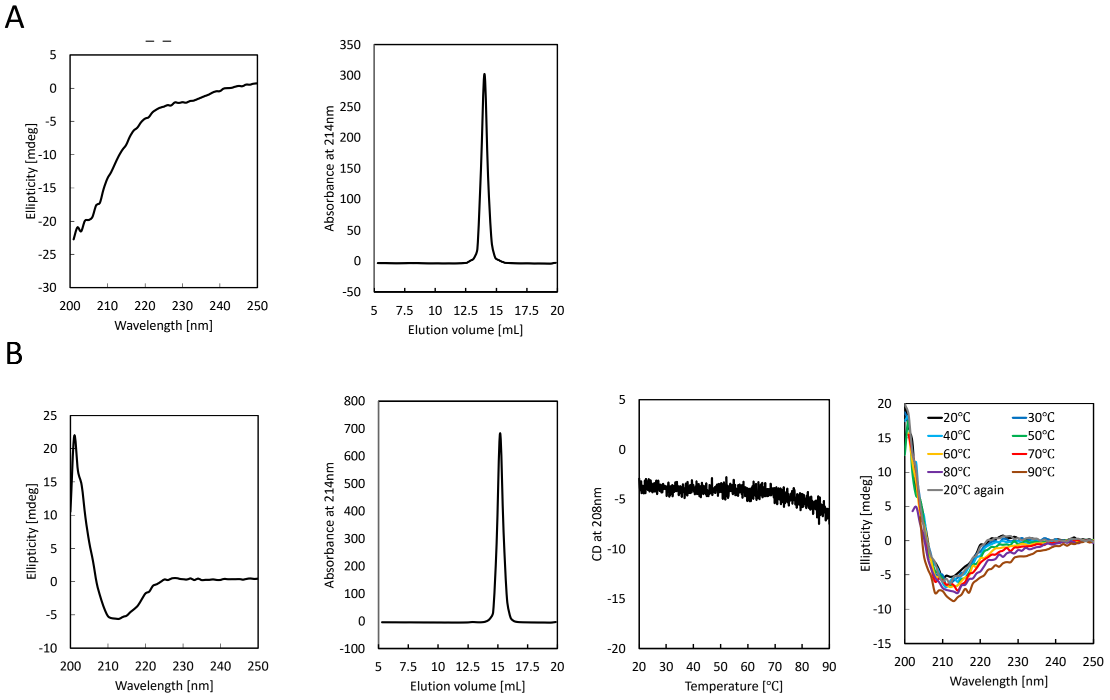

Supplementary figure 19. Experimental characterizations of the reconstructed SH3-fold proteins. Size exclusion chromatography, CD spectra, denaturation curves, and comparisons of CD spectra at different temperatures (20, 30, 40, 50, 60, 70, 80, 90, 20°C again (refold)) for (A) design-23 and (B) design-24. Source data are provided as a Source Data file.

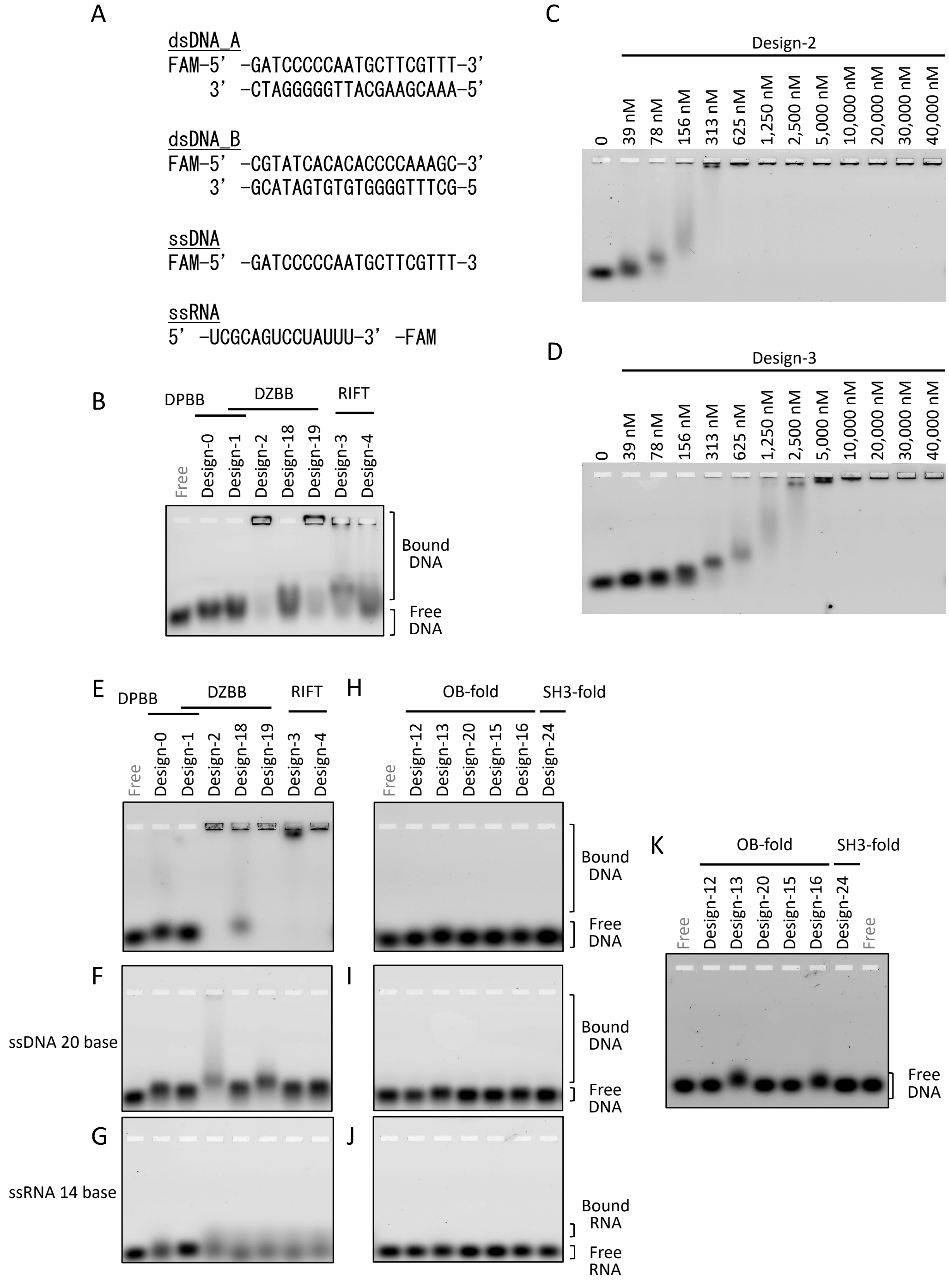

Supplementary figure 20. (A) The sequences of fluorescently-labeled oligo-nucleotides used in EMSA experiments. (B) EMSA results of the homo-dimeric DPBB, DZBB, and RIFT proteins with dsDNA_A in the presence of 500 mM NaCl. (C and D) EMSA results with increasing protein concentration of design-**2** and design-**3.** (E–G) EMSA results of DPBB, DZBB, and RIFT proteins with (E) dsDNA_B, (F) ssDNA, and (G) ssRNA. (H–J) EMSA results of the OB and SH3 proteins with (H) dsDNA_A, (I) ssDNA, and (J)

ssRNA. (K) EMSA results with higher concentrations of OB- and SH3-fold proteins (40 μM). Source data are provided as a Source Data file.

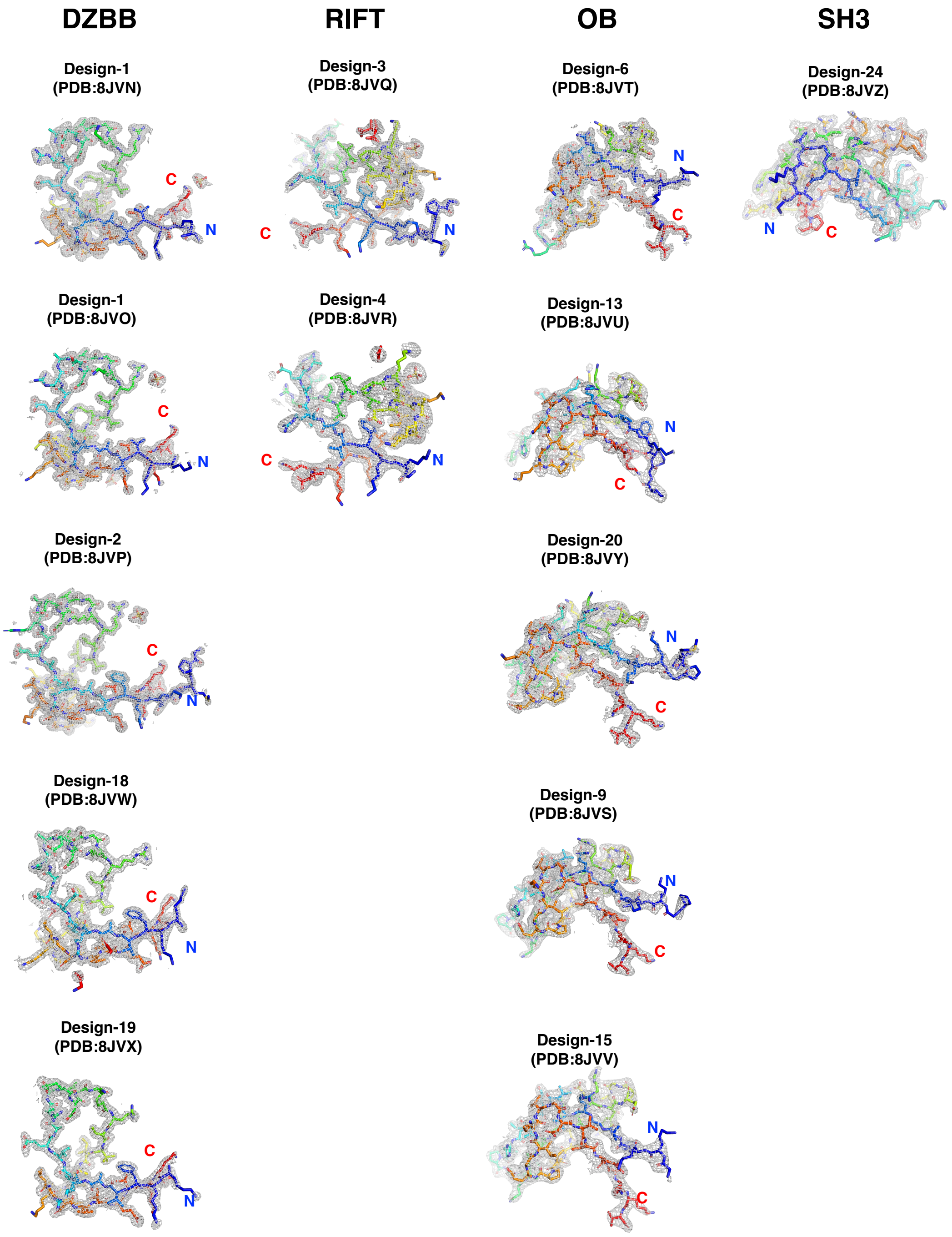

Supplementary figure 21. The density map of each designed protein. The monomer chains were depicted with their density map contoured at 1σ.

Supplementary table 1. Mutant sequences and information.

Fold	Design No.	Mutant name	Amino acid sequence	Template mutant	Length	Mass	pI
DPBB	Design-0	mk2h_dMILPS	GPMPGKKVVARVAEAYAEDVGKRVVRVDKYERAKVGVKVGDYVEVKKV		48(43)	5274.2	9.8
DPBB/DZBB	Design-1	mk2h_dMILPYS	GPMPGKKVVARVAEARAEDVGKRVVRVDKAERAKVGVKVGDVVEVKKV	mk2h_dMILPS	48(43)	5111.1	10.2
DZBB	Design-2	Ph1	GPMPGKKFVARVAEARAEDVGKRVVIIPKAERAKVGIKVGDVVEVKKV	mk2h_dMILPYS	48(43)	5126.2	10.2
RIFT	Design-3	Ph1_DG	GPMPGKKFVARVAEADGKRVVIIPKAERAKVGIKVGDVVEVKKV	Ph1	44(39)	4670.7	10.2
	Design-4	Ph1_GG	GPMPGKKFVARVAEAGGKRVVIIPKAERAKVGIKVGDVVEVKKV	Ph1	44(39)	4612.6	10.5
	Design-5	Ph1_GD	GPMPGKKFVARVAEAGDKRVVIIPKAERAKVGIKVGDVVEVKKV	Ph1	44(39)	4670.7	10.2
OB	Design-6	tkoL2_v1	GPMPGKKVVARVEEILHDPGRTAPVARVKFEDGTKKLIIAPEGVKVGDVVEVKKV	mk2h_dMILPYS + rProtein L2(*T. kodakarensis*)	55(50)	5889.0	9.5
	Design-7	tkoL2_v2	GPMPGKKVVARVEEILHDPGRTAPVARVKFEDGTKKLIDKAERAKVGVKVGDVVEVKKV	mk2h_dMILPYS + rProtein L2(*T. kodakarensis*)	59(54)	6376.6	9.8
	Design-8	tkoL2_v3	GPMPGKKVVARVEEILHDPGRTAPVARVKFEDGTKKLIRVDKAERAKVGVKVGDVVEVKKV	mk2h_dMILPYS + rProtein L2(*T. kodakarensis*)	61(56)	6631.9	10.0
	Design-9	mkaL2_v1	GPMPGKKVVARVVELLHDPARNAPVARVRFEDGEERLILVPEGVKVGDVVEVKKV	mk2h_dMILPYS + rProtein L2(*M. kandrerii*)	55(50)	5999.1	8.4
	Design-10	mkaL2_v2	GPMPGKKVVARVVELLHDPARNAPVARVRFEDGEERLIDKAERAKVGVKVGDVVEVKKV	mk2h_dMILPYS + rProtein L2(*M. kandrerii*)	59(54)	6458.6	9.6
	Design-11	mkaL2_v3	GPMPGKKVVARVVELLHDPARNAPVARVRFEDGEERLIRVDKAERAKVGVKVGDVVEVKKV	mk2h_dMILPYS + rProtein L2(*M. kandrerii*)	61(56)	6713.9	9.9
	Design-12	tkoL2_v1.1	GPMPGKKFVARVEEILHDPGRTAPVARVKFEDGTKKLIIAPEGIKVGDVVEVKKV	tkoL2_v1	55(50)	5951.1	9.5
	Design-13	tkoL2_v1.2	GPMPGKKFVARVEEILHDPGRTAPVARVKFEDGTKRVVIIPKGIKVGDVVEVKKV	tkoL2_1_v1.1	55(50)	5992.2	10.0
	Design-14	tkoL2_v1.3	GPMPGKKFVARVEEILHDPGRTAPVARARAEDVGKRVVIIPKGIKVGDVVEVKKV	tkoL2_1_v1.2	55(50)	5914.1	10.1
	Design-15	mkaL2_v1.1	GPMPGKKFVARVVELLHDPARNAPVARVRFEDGEERLILVPEGIKVGDVVEVKKV	mkaL2_v1	55(50)	6061.2	8.4
	Design-16	mkaL2_v1.2	GPMPGKKFVARVVELLHDPARNAPVARVRFEDGEKRVVIIPKGIKVGDVVEVKKV	mkaL2_v1.1	55(50)	6045.2	10.1
	Design-17	mkaL2_v1.3	GPMPGKKFVARVVELLHDPARNAPVARARAEDVGKRVVIIPKGIKVGDVVEVKKV	mkaL2_v1.2	55(50)	5911.1	10.4
DZBB	Design-18	tkoL2_v1.2_Z	GPMPGKKFVARVEEARAEDVGKRVVIIPKGIKVGDVVEVKKV	tkoL2_v1.2	42(37)	4529.5	9.9
	Design-19	mkaL2_v1.2_Z	GPMPGKKFVARVVEARAEDVGKRVVIIPKGIKVGDVVEVKKV	mkaL2_v1.2	42(37)	4499.5	10.1
OB	Design-20	tkoL2_v1_turn	GPMPGKKVVARVEEILDGVARVKFEDGTKKLIIAPEGVKVGDVVEVKKV	tkoL2_v1	49(44)	5229.3	9.2
	Design-21	tkoL2_v1.2_turn	GPMPGKKFVARVEEILDGVARVKFEDGTKRVVIIPKGIKVGDVVEVKKV	tkoL2_v1.2	49(44)	5332.4	9.8
	Design-22	mkaL2_v1.2_turn	GPMPGKKFVARVVELLDGVARVRFEDGEKRVVIIPKGIKVGDVVEVKKV	mkaL2_v1.2	49(44)	5358.5	9.9
SH3	Design-23	tkoL2_v1_SH3	GPGVKVGDVVEVKKDGKKVVARVEEILHDPGRTAPVARVKFEDGTKKLIIAPE	tkoL2_v1	54(49)	5775.8	9.2
	Design-24	mkaL2_v1_SH3	GPGVKVGDVVEVKKDGKKVVARVVELLHDPARNAPVARVRFEDGEERLILVPE	mkaL2_v1	54(49)	5885.9	6.8

Supplementary table 2. Synthetic DNA sequences and primers.

Protein Name	Sequence
Ph1	AAGTCCTCTTTCAGGGACCCATGCCTGGCAAAAAGTTTGTTGCACGTGTTGCCGAAGCACGTGCCGAAGATGTTGGTAAACGTGTTGTTAT<br>TATTCCGAAAGCAGAACGTGCCAAAGTGGGTATTAAAGTTGGTGATGTTGTGGAAGTGAAGAAAGTGTAAGGATCCGAATTCTGTACAGG
tkoL2_v1	AAGTCCTCTTTCAGGGACCCATGCCTGGTAAAAAGGTTGTTGCACGTGTTGAAGAAATTCTGCATGATCCGGGTCGTACCGCACCGGTTGC<br>GCGTGTTAAATTTGAAGATGGCACCAAAAAGCTGATTATTGCACCGGAAGGTGTTAAAGTTGGTGATGTTGTGGAAGTTGGTCCGGAAGCA<br>CCGATTGCAATTGGTAATACCCTGCCGTAAGGATCCGAATTCTGTACAGG
tkoL2_v2	AAGTCCTCTTTCAGGGACCCATGCCTGGTAAAAAGGTTGTTGCACGTGTTGAAGAAATTCTGCATGATCCGGGTCGTACCGCACCGGTTGC<br>GCGTGTTAAATTTGAAGATGGCACCAAAAAGCTGATCGATAAAGCAGAACGTGCAAAAGTTGGTGTTAAAGTTGGTGATGTTGTGGAAGTT<br>GGTCCGGAAGCACCGATTGCAATTGGTAATACCCTGCCGTAAGGATCCGAATTCTGTACAGG
tkoL2_v3	AAGTCCTCTTTCAGGGACCCATGCCTGGTAAAAAGGTTGTTGCACGTGTTGAAGAAATTCTGCATGATCCGGGTCGTACCGCACCGGTTGC<br>GCGTGTTAAATTTGAAGATGGCACCAAAAAGCTGATCCGTGTTGATAAAGCAGAACGTGCAAAAGTTGGTGTTAAAGTTGGTGATGTTGTG<br>GAAGTTGGTCCGGAAGCACCGATTGCAATTGGTAATACCCTGCCGTAAGGATCCGAATTCTGTACAGG
tkoL2_v1.1	AAGTCCTCTTTCAGGGACCCATGCCTGGCAAAAAGTTTGTTGCACGTGTTGAAGAAATTCTGCATGATCCGGGTCGTACCGCACCGGTTGC<br>GCGTGTTAAATTTGAAGATGGCACCAAAAAGCTGATTATTGCACCGGAAGGTATTAAAGTTGGTGATGTTGTTGAAGTGAAAAAGGTGTAA<br>GGATCCGAATTCTGTACAGG
tkoL2_v1.2	AAGTCCTCTTTCAGGGACCCATGCCTGGCAAAAAGTTTGTTGCACGTGTTGAAGAAATTCTGCATGATCCGGGTCGTACCGCACCGGTTGC<br>GCGTGTTAAATTTGAAGATGGCACCAAACGTGTTGTGATTATTCCGAAAGGTATCAAAGTTGGTGATGTGGTGGAAGTGAAAAAGGTGTAA<br>GGATCCGAATTCTGTACAGG
tkoL2_v1.3	AAGTCCTCTTTCAGGGACCCATGCCTGGCAAAAAGTTTGTTGCACGTGTTGAAGAAATTCTGCATGATCCGGGTCGTACCGCACCGGTTGC<br>GCGTGCACGTGCCGAAGATGTTGGTAAACGTGTTGTTATTATTCCGAAAGGCATCAAAGTTGGTGATGTTGTGGAAGTGAAAAAGGTGTAA<br>GGATCCGAATTCTGTACAGG
tkoL2_v1_turn	AAGTCCTCTTTCAGGGACCCATGCCTGGTAAAAAGGTTGTTGCACGTGTTGAAGAAATTCTGGATGGTGTTGCCCGTGTGAAATTTGAAGA<br>TGGCACCAAAAAGCTGATTATTGCACCGGAAGGTGTTAAAGTTGGTGATGTTGTTGAAGTGAAGAAAGTGTAAGGATCCGAATTCTGTACA<br>GG
mkaL2_v1	AAGTCCTCTTTCAGGGACCCATGCCTGGTAAAAAGGTTGTTGCACGTGTTGTTGAACTGCTGCATGATCCGGCACGTAATGCACCGGTTGC<br>GCGTGTTCGTTTTGAAGATGGTGAAGAACGTCTGATTCTGGTTCCGGAAGGTGTTAAAGTTGGTGATGTTGTGGAAGTTGGTGTTAGCGCA<br>GAAATCAAACCGGGTAATACCCTGCCGTAAGGATCCGAATTCTGTACAGG
mkaL2_v2	AAGTCCTCTTTCAGGGACCCATGCCTGGTAAAAAGGTTGTTGCACGTGTTGTTGAACTGCTGCATGATCCGGCACGTAATGCACCGGTTGC<br>GCGTGTTCGTTTTGAAGATGGTGAAGAACGTCTGATTGATAAAGCAGAACGTGCAAAAGTTGGTGTGAAAGTTGGTGATGTTGTGGAAGTT<br>GGCGTTAGCGCAGAAATCAAACCGGGTAATACCCTGCCGTAAGGATCCGAATTCTGTACAGG
mkaL2_v3	AAGTCCTCTTTCAGGGACCCATGCCTGGTAAAAAGGTTGTTGCACGTGTTGTTGAACTGCTGCATGATCCGGCACGTAATGCACCGGTTGC<br>GCGTGTTCGTTTTGAAGATGGTGAAGAACGTCTGATTCGTGTTGATAAAGCAGAACGTGCAAAAGTTGGTGTTAAAGTTGGTGATGTTGTG<br>GAAGTTGGTGTTAGCGCAGAAATCAAACCGGGTAATACCCTGCCGTAAGGATCCGAATTCTGTACAGG
mkaL2_v1.1	AAGTCCTCTTTCAGGGACCCATGCCTGGCAAAAAGTTTGTTGCACGTGTTGTTGAACTGCTGCATGATCCGGCACGTAATGCACCGGTTGC<br>GCGTGTTCGTTTTGAAGATGGTGAAGAACGTCTGATTCTGGTTCCGGAAGGTATTAAAGTTGGTGATGTTGTGGAAGTGAAGAAAGTGTAA<br>GGATCCGAATTCTGTACAGG
mkaL2_v1.2	AAGTCCTCTTTCAGGGACCCATGCCTGGCAAAAAGTTTGTTGCACGTGTTGTTGAACTGCTGCATGATCCGGCACGTAATGCACCGGTTGC<br>GCGTGTTCGTTTTGAAGATGGTGAAAAACGTGTTGTGATTATCCCGAAAGGTATCAAAGTTGGTGATGTTGTGGAAGTGAAAAAGGTGTAA<br>GGATCCGAATTCTGTACAGG
mkaL2_v1.3	AAGTCCTCTTTCAGGGACCCATGCCTGGCAAAAAGTTTGTTGCACGTGTTGTTGAACTGCTGCATGATCCGGCACGTAATGCACCGGTTGC<br>GCGTGCACGTGCCGAAGATGTTGGTAAACGTGTTGTGATTATTCCGAAAGGCATTAAAGTTGGTGATGTGGTGGAAGTGAAAAAGGTGTAA<br>GGATCCGAATTCTGTACAGG
tkoL2_v1.2_Z	AAGTCCTCTTTCAGGGACCCATGCCTGGCAAAAAGTTTGTTGCACGTGTTGAAGAGGCACGTGCGGAAGATGTTGGTAAACGTGTTGTTAT<br>TATTCCGAAAGGCATCAAAGTTGGTGATGTTGTGGAAGTGAAAAAGGTGTAAGGATCCGAATTCTGTACAGG
mkaL2_v1.2_Z	AAGTCCTCTTTCAGGGACCCATGCCTGGCAAAAAGTTTGTTGCACGTGTTGTTGAAGCACGTGCCGAAGATGTTGGTAAACGTGTTGTGAT<br>TATTCCGAAAGGCATTAAAGTTGGTGATGTGGTGGAAGTGAAAAAGGTGTAAGGATCCGAATTCTGTACAGG
tkoL2_v1_SH3	AAGTCCTCTTTCAGGGACCCGGTGTTAAAGTTGGTGATGTTGTGGAAGTGAAAAAGGATGGTAAAAAGGTTGTTGCCCGTGTGGAAGAAAT<br>TCTGCATGATCCGGGTCGTACCGCACCGGTTGCACGTGTTAAATTTGAAGATGGCACCAAGAAACTGATTATCGCACCGGAATAAGGATCC<br>GAATTCTGTACAGG
mkaL2_v1_SH3	AAGTCCTCTTTCAGGGACCCGGTGTTAAAGTTGGTGATGTTGTGGAAGTGAAAAAGGATGGTAAAAAGGTTGTTGCCCGTGTTGTTGAACT<br>GCTGCATGATCCGGCACGTAATGCACCGGTTGCACGTGTTCGTTTTGAAGATGGTGAAGAACGTCTGATTCTGGTTCCGGAATAAGGATCC<br>GAATTCTGTACAGG
Primers	
Cloning_upstream	AAGTCCTCTTTCAGGGACCC
Cloning_downstream	CCTGTACAGAATTCGGATCC
pET47b_up	GGGTCCCTGAAAGAGGACTT
pET47b_down	GGATCCGAATTCTGTACAGG
Ph1_DG	CGTGTTGCCGAAGCAGATGGTAAACGTGTTGTTATTATTCCG
Ph1_DG_comp	CGGAATAATAACAACACGTTTACCATCTGCTTCGGCAACACG
Ph1_GG	CGTGTTGCCGAAGCAGGTGGCAAACGTGTTGTTATTATTCCG
Ph1_GG_comp	CGGAATAATAACAACACGTTTGCCACCTGCTTCGGCAACACG
Ph1_GD	CGTGTTGCCGAAGCAGGTGATAAACGTGTTGTTATTATTCCG
Ph1_GD_comp	CGGAATAATAACAACACGTTTATCACCTGCTTCGGCAACACG

Supplementary table S3. Summary of biophysical experiment for all protein mutants.

Fold	Design No.	Solubility	CD spectrum (500mM Ammonium sulfate)	Aparent molecular masses	Estimated oligomeric state	Fold in the crystal structure (pdbid)
DPBB/DZBB	Design-1	Yes	Random coil	nd	nd	DPBB (7DXY, 7DXZ) DZBB (8JVN, 8JVO)
DZBB	Design-2	Yes	Partially fold (fold)	15282.7	3.0	DZBB (8JVP)
RIFT	Design-3	Yes	Partially fold (fold)	nd	nd	RIFT (8JVQ)
	Design-4	Yes	Partially fold (fold)	nd	nd	RIFT (8JVR)
	Design-5	Yes	Partially fold (fold)	nd	nd	
OB	Design-6	Yes	Fold	12761.8	2.2	OB (8JVT)
	Design-7	Yes	Random coil	26245.9	4.1	
	Design-8	Yes	Random coil	29243.8	4.4	
	Design-9	Yes	Fold	10279.4	1.7	OB (8JVS)
	Design-10	Yes	Random coil	23555.3	3.6	
	Design-11	Yes	Random coil	17653.4	2.6	
	Design-12	Yes	Fold	10656.7	1.8	
	Design-13	Yes	Fold	10279.4	1.7	OB (8JVU)
	Design-14	Yes	Random coil	25316.5	4.3	
	Design-15	Yes	Fold	9915.4	1.6	OB (8JVV)
	Design-16	Yes	Fold	11048.0	1.8	
	Design-17	Yes	Random coil	nd	nd	
DZBB	Design-18	Yes	Fold	13230.3	2.9	DZBB (8JVW)
	Design-19	Yes	Fold	13230.3	2.9	DZBB (8JVX)
OB	Design-20	Yes	Fold	10656.7	2.0	OB (8JVY)
	Design-21	Yes	Fold	9502.4	1.8	
	Design-22	Yes	Fold	13617.4	2.5	
SH3	Design-23	Yes	Random coil	13602.7	1.6	
	Design-24	Yes	Fold	8940.718543	2.3	SH3 (8JVZ)

Supplementary table 4. Summary of crystallization methods.

	Concentration [mg/mL]	Conditions	Date	Site	Beam line	MR model
mk2h_ΔMILPYS (DZBB fold)	7.6	40% PEG400, 100mM Tris pH 8.5, 200mM Lithium sulfate	200910	SLS	X06SA	AF2 model of Ph1 (RIFT fold)
mk2h_ΔMILPYS (DZBB fold; synthesized peptide)	16.8	40% PEG400, 100mM Tris pH 8.5, 200mM Lithium sulfate	221012	PF	BL5A	mk2h_ΔMILPYS (DZBB fold)
Ph1	3.2	30% PEG400, 100mM Tris pH 8.0, 400mM Lithium sulfate	220127	SPring-8	Bl26B2	AF2 model of Ph1 (RIFT fold)
Ph1_DG	15.8	3200mM Ammonium sulfate, 100mM Citric acid/Sodium hydroxide pH 5.0	220422	SPring-8	Bl26B2	Ph1
Ph1_GG	12.5	50% PEG400, 100mM Sodium acetate/Acetic acid pH 4.5, 200mM Lithium sulfate	220707	PF	BL17A	Ph1_lDG
mkaL2_v1	8.3	20%PEG_6000, 100mM Tris pH 8.0, 200mM Lithium chloride	220707	PF	BL17A	AF2 model of mkaL2_v1
tkoL2_v1	82.9	2% PEG400, 100mM Tris pH 8.5, 2000mM Lithium sulfate	220707	PF	BL17A	AF2 model of tkoL2_v1
tkoL2_v1.2	34.8	30% 2-propanol, 100mM Tris-HCl pH 8.5, 30% PEG3350	221012	PF	BL5A	tkoL2_v1
mkaL2_v1.1	17.7	30% jeffamin ED-2001, 100mM HEPES pH 7.0	221012	PF	BL5A	mkaL2_v1
tkoL2_v1.2_Z	7.4	70% MPD, 100mM HEPES pH 7.0	221012	PF	BL5A	Ph1
mkaL2_v1.2_Z	1.6	30% 2-propanol, 100mM Tris-HCl pH 8.5, 30% PEG3350	230223	PF	BL1A	tkoL2_v1.2_Z
tkoL2_v1_turn	27.8	20% PEG3350, 200mM Ammonium citrate tribasic pH 7.5	221012	PF	BL5A	tkoL2_v1
mkaL2_v1_SH3	9.6	30% 2-propanol, 100mM Tris-HCl pH 8.5, 30% PEG3350	221012	PF	BL5A	mkaL2_v1

Supplementary table S5. Data collection and refinement statistics.

	mk2h_dMILPYS (Bacteria produced) 8JVN	mk2h_dMILPYS (Synthetic peptide) 8JVO	Ph1 8JVP	Ph1_DG 8JVQ	Ph1_GG 8JVR	tkoL2_v1 8JVS
**Data collection**						
Space group	P3(2)21	P3(2)21	P3(2)21	P3(2)21	P3(2)21	P4(1)2(1)2
Cell dimensions						
a, *b*, *c* (Å)	34.1, 34.1, 54.3	33.7, 33.7, 54.8	34.9, 34.9, 54.7	35.0, 35.0, 54.6	34.5, 34.5, 55.1	40.5, 40.5, 68.6
α, β, γ (°)	90.0, 90.0, 120.0	90.0, 90.0, 120.0	90.0, 90.0, 120.0	90.0, 90.0, 120.0	90.0, 90.0, 120.0	90.0, 90.0, 90.0
Resolution (Å) *	50-1.4 (1.5-1.4)	50-1.7 (1.8-1.7)	50-1.3 (1.4-1.3)	50-1.4 (1.5-1.4)	50-1.8 (1.9-1.8)	50-1.5 (1.59-1.5)
R_{sym} or R_{merge}*	3.1 (24.8)	4.4 (16.2)	4.0 (31.8)	3.1 (32.5)	4.0 (74.8)	8.5 (36.4)
$I / \sigma I$*	19.13 (3.37)	29.2 (7.3)	42.48 (8.64)	54.24 (10.11)	16.7 (2.2)	17.6 (4.7)
Completeness (%)*	97.0 (94.9)	99.7 (98.5)	98.7 (99.2)	99.2 (99.8)	98.5 (96.7)	100 (99.9)
Redundancy*	4.2 (4.9)	8.7 (7.6)	18.2 (18.2)	20.1 (20.6)	5.2 (4.9)	12.6 (12.3)
**Refinement**						
Resolution (Å)	29.5-1.4	29.2-1.7	20.4-1.3	26.5-1.4	29.8–1.8	34.8-1.5
No. reflections*	7291 (1137)	4271 (657)	9854 (1560)	7958 (1249)	3764 (594)	9673 (1509)
R_{work} / R_{free}	0.1905 / 0.2108	0.1969 / 0.2136	0.1897 /0.2247	0.2123 /0.2394	0.2308 /0.2429	0.1992 /0.2101
No. atoms	379	384	428	393	320	435
Protein	332	328	376	335	305	384
Ligand/ion	15	15	5	18	5	0
Water	32	41	47	40	10	51
B-factors	36.25	26.80	26.63	31.01	54.94	34.20
Protein	34.10	24.69	25.14	28.62	54.49	32.71
Ligand/ion	66.80	53.45	30.53	55.46	73.27	-
Water	44.18	33.85	38.17	40.08	53.05	45.42
R.m.s. deviations						
Bond lengths	0.015	0.016	0.012	0.017	0.013	0.007
Bond angles (°)	1.254	1.55	1.205	1.28	1.575	1.13

*Values in parentheses are for highest-resolution shell.

	mkaL2_v1	mkaL2_v1.1	tkoL2_v1.2	tkoL2_v1.2_Z	mkaL2_v1.2_Z	tkoL2_v1_turn	mkaL2_v1_SH3
	8JVT	8JVV	8JVU	8JVW	8JVX	8JVY	8JVZ
**Data collection**							
Space group	C2	P2	P6(1)	I222	P2(1)2(1)2(1)	P6(5)22	P2(1)2(1)2
Cell dimensions							
a, *b*, *c* (Å)	123.7, 53.6, 55.4	56.2, 95.2, 64.4	32.7, 32.7, 75.9	23.9, 44.3, 83.9	34.4, 37.7, 52.2	58.9, 58.9, 54.6	28.3, 38.9, 90.4
α, β, γ (°)	90.0, 113.4,	90.0, 114.1, 90.0	90.0, 90.0, 60.0	90.0, 90.0, 90.0	90.0, 90.0, 90.0	90.0, 90.0, 120.0	89.8, 89.8, 90.1
Resolution (Å) *	50-2.1 (2.2-2.1)	50-2.3 (2.44-2.3)	50-2.5 (2.7-2.5)	50-1.8 (1.9-1.8)	50-1.8 (1.9-1.8)	50-1.9 (2.0-1.9)	50–1.9 (2.0-1.9)
R_{sym} or R_{merge}*	4.6 (80.3)	4.7 (63.7)	8.4 (163.9)	5.6 (82.2)	6.9 (54.0)	4.4 (73.9)	7.5 (62.8)
I / σI*	10.7(1.0)	17.85 (2.0)	17.67 (1.73)	18.77 (2.61)	13.95 (3.27)	43.31 (4.75)	13.58 (2.64)
Completeness (%)*	98.6 (98.3)	99.2 (98.2)	100 (100)	99.4 (98.7)	99.2 (98.4)	99.7 (99.5)	99.6 (98.5)
Redundancy*	3.0 (3.0)	3.38 (3.27)	10.3 (10.6)	6.2 (6.4)	6.4 (6.7)	18.2 (18.1)	6.2 (6.0)
**Refinement**							
Resolution (Å)	38.7-2.1	47.7-2.3	28.3-2.5	42.1-1.8	30.6-1.8	37.2-1.9	38.8-1.9
No. reflections*	19517(3120)	27556 (4391)	1594 (242)	4385 (678)	6620 (1023)	4761 (736)	8381 (1309)
R_{work} / R_{free}	0.2044 / 0.2471	0.2287 / 0.2523	0.2432 / 0.2665	0.2114 / 0.2305	0.2028 / 0.2175	0.2257 / 0.2452	0.2188 / 0.2321
No. atoms	2420	4845	369	328	641	399	881
Protein	2397	4735	368	288	598	371	774
Ligand/ion	0	0	0	15	0	0	0
Water	23	110	1	25	43	28	107
B-factors	83.15	57.71	69.42	39.31	32.36	54.79	36.00
Protein	83.27	57.76	69.45	39.06	32.08	55.02	35.60
Ligand/ion	-	-	-	27.82	-	-	-
Water	70.57	55.82	60.25	42.40	36.22	51.73	38.90
R.m.s. deviations							
Bond lengths (Å)	0.01	0.016	0.018	0.024	0.014	0.012	0.013
Bond angles (°)	1.267	1.621	2.106	1.855	1.47	1.328	1.478

*Values in parentheses are for highest-resolution shell.

Supplementary References

(1) Jumper, J.; Evans, R.; Pritzel, A.; Green, T.; Figurnov, M.; Ronneberger, O.; Tunyasuvunakool, K.; Bates, R.; Žídek, A.; Potapenko, A.; Bridgland, A.; Meyer, C.; Kohl, S. A. A.; Ballard, A. J.; Cowie, A.; Romera-Paredes, B.; Nikolov, S.; Jain, R.; Adler, J.; Back, T.; Petersen, S.; Reiman, D.; Clancy, E.; Zielinski, M.; Steinegger, M.; Pacholska, M.; Berghammer, T.; Bodenstein, S.; Silver, D.; Vinyals, O.; Senior, A. W.; Kavukcuoglu, K.; Kohli, P.; Hassabis, D. Highly Accurate Protein Structure Prediction with AlphaFold. *Nature* **2021**, *596* (7873), 583–589.

(2) Coles, M.; Hulko, M.; Djuranovic, S.; Truffault, V.; Koretke, K.; Martin, J.; Lupas, A. N. Common Evolutionary Origin of Swapped-Hairpin and Double-Psi Beta Barrels. *Structure* **2006**, *14* (10), 1489–1498.

(3) Yagi, S.; Padhi, A. K.; Vucinic, J.; Barbe, S.; Schiex, T.; Nakagawa, R.; Simoncini, D.; Zhang, K. Y. J.; Tagami, S. Seven Amino Acid Types Suffice to Create the Core Fold of RNA Polymerase. *J. Am. Chem. Soc.* **2021**, *143* (39), 15998–16006.

(4) Yadid, I.; Kirshenbaum, N.; Sharon, M.; Dym, O.; Tawfik, D. S. Metamorphic Proteins Mediate Evolutionary Transitions of Structure. *Proc. Natl. Acad. Sci. U. S. A.* **2010**, *107* (16), 7287–7292.

(5) Dishman, A. F.; Volkman, B. F. Unfolding the Mysteries of Protein Metamorphosis. *ACS Chem. Biol.* **2018**, *13* (6), 1438–1446.

(6) Dishman, A. F.; Tyler, R. C.; Fox, J. C.; Kleist, A. B.; Prehoda, K. E.; Babu, M. M.; Peterson, F. C.; Volkman, B. F. Evolution of Fold Switching in a Metamorphic Protein. *Science* **2021**, *371* (6524), 86–90.

(7) Liu, Z.; Rossi, J.-C.; Pascal, R. How Prebiotic Chemistry and Early Life Chose Phosphate. *Life* **2019**, *9* (1). https://doi.org/10.3390/life9010026.

(8) Leustek, T. Sulfate Metabolism. *Arabidopsis Book* **2002**, *1*, e0017.

(9) Giacobelli, V. G.; Fujishima, K.; Lepšík, M.; Tretyachenko, V.; Kadavá, T.; Makarov, M.; Bednárová, L.; Novák, P.; Hlouchová, K. In Vitro Evolution Reveals Non-Cationic Protein-RNA Interaction Mediated by Metal Ions. *Mol. Biol. Evol.* **2022**, *39* (3), msac032.

(10) Despotović, D.; Longo, L. M.; Aharon, E.; Kahana, A.; Scherf, T.; Gruic-Sovulj, I.; Tawfik, D. S. Polyamines Mediate Folding of Primordial Hyperacidic Helical Proteins. *Biochemistry* **2020**, *59* (46), 4456–4462.

(11) Longo, L. M.; Lee, J.; Blaber, M. Simplified Protein Design Biased for Prebiotic Amino Acids Yields a Foldable, Halophilic Protein. *Proc. Natl. Acad. Sci. U. S. A.* **2013**, *110* (6), 2135–2139.

(12) Alva, V.; Koretke, K. K.; Coles, M.; Lupas, A. N. Cradle-Loop Barrels and the Concept of Metafolds in Protein Classification by Natural Descent. *Curr. Opin. Struct. Biol.* **2008**, *18* (3), 358–365.

(13) Lupas, A. N.; Koretke, K. K. Evolution of Protein Folds. *Computational structural biology: methods and applications* **2008**, 131–152.

(14) Aubel, M.; Bornberg-Bauer, E. Introducing Creative Destruction as a Mechanism in Protein Evolution. *Proc. Natl. Acad. Sci. U. S. A.* **2023**, *120* (6), e2220460120.

(15) Alvarez-Carreño, C.; Gupta, R. J.; Petrov, A. S.; Williams, L. D. Creative Destruction: New Protein Folds from Old. *Proc. Natl. Acad. Sci. U. S. A.* **2022**, *119* (52), e2207897119.

(16) Alvarez-Carreno, C.; Penev, P. I.; Petrov, A. S.; Williams, L. D. Fold Evolution

before LUCA: Common Ancestry of SH3 Domains and OB Domains. *Mol. Biol. Evol.* **2021**, *38* (11), 5134–5143.

(17) Agrawal, V.; Kishan, R. K. Functional Evolution of Two Subtly Different (Similar) Folds. *BMC Struct. Biol.* **2001**, *1*, 5.

(18) Theobald, D. L.; Mitton-Fry, R. M.; Wuttke, D. S. Nucleic Acid Recognition by OB-Fold Proteins. *Annu. Rev. Biophys. Biomol. Struct.* **2003**, *32*, 115–133.

(19) Alva, V.; Dunin-Horkawicz, S.; Habeck, M.; Coles, M.; Lupas, A. N. The GD Box: A Widespread Noncontiguous Supersecondary Structural Element. *Protein Sci.* **2009**, *18* (9), 1961–1966.
